# Antimicrobial activities of actinomycetes isolated from unexplored regions of Sundarbans mangrove ecosystem

**DOI:** 10.1186/s12866-015-0495-4

**Published:** 2015-08-21

**Authors:** Sohan Sengupta, Arnab Pramanik, Abhrajyoti Ghosh, Maitree Bhattacharyya

**Affiliations:** Department of Biochemistry, University of Calcutta, 35, Ballygunge Circular Road, Kolkata, 700 019 India; Department of Biochemistry, Bose Institute, Centenary Campus, P 1/12, C.I.T Road, Scheme VIIM, Kolkata, 700 054 West Bengal India

## Abstract

**Background:**

New broad spectrum antimicrobial agents are urgently needed to combat frequently emerging multi drug resistant pathogens. Actinomycetes, the most talented group of microorganisms isolated from unexplored regions of the world may be the ultimate solution to this problem. Thus the aim of this study was to isolate several bioactive actinomycetes strains capable of producing antimicrobial secondary metabolite from Sundarbans, the only mangrove tiger land of the world.

**Results:**

Fifty four actinomycetes were isolated and analyzed for antimicrobial activity against fifteen test organisms including three phytopathogens. Nine morphologically distinct and biologically active isolates were subjected to polyphasic identification study.16 s rDNA sequencing indicated eight isolates to reveal maximum similarity to the genus streptomyces, whereas one isolate presented only 93.57 % similarity with *Streptomyces albogriseolus* NRRL B-1305^T^. Seventy-one carbon sources and twenty-three chemical sources utilization assay revealed their metabolic relatedness. Among these nine isolates three specific strains were found to have notably higher degree of antimicrobial potential effective in a broader range including phyto-pathogenic fungus. Finally the strain SMS_SU21, which showed antimicrobial activity with MIC value of 0.05 mg ml^−1^ and antioxidant activity with IC50 value of 0.242 ± 0.33 mg ml^−1^ was detected to be the most potential one. True prospective of this strain was evaluated utilizing GC-MS and the bioactive compound responsible for antimicrobial activity was purified.

**Conclusion:**

Rare bioactive actinomycetes were isolated from unexplored heritage site. Antimicrobial compound has also been identified and purified which is active against a broad range of pathogens.

**Electronic supplementary material:**

The online version of this article (doi:10.1186/s12866-015-0495-4) contains supplementary material, which is available to authorized users.

## Background

Mangroves are the tidal forest existing in the intertidal zone of sheltered shores, estuarine tidal creek, lagoon, and marshes mudflats of the tropical and subtropical regions of the world. Mangrove ecosystem supports a wide range of living resources and protects the coastal zone. Sundarbans, being the largest mangrove delta of the world, is unique for its ecological dynamics and is routinely inundated with low, moderate or high saline water. This ecosystem is ideally situated at the interphase between the terrestrial and marine environment and harbors a rich and diverse group of microorganisms [1]. Due to its high index of species diversity and limited consumable nutrient sources, an interspecies competition is an obvious phenomenon within the residing microbial population. Several secondary metabolites (such as dipterocarp resins) from plant origin are available which are toxic to microorganisms, need to be degraded or detoxified by certain microbes for their own sustenance. In such an ecosystem, the microbes are under the selection pressure and ultimately evolve to produce novel bioactive secondary metabolites as one of their survival strategies. In the microbial world, actinomycetes are phylogenetically defined as a taxa within the high G + C content subdivision of the Gram-positive phylum [2], involved in important processes in a wide range of habitats [3] and holds the prime position in production of bioactive metabolites. They are responsible for the production of almost half of the discovered bioactive secondary metabolites [4], notably antitumor agents [5], immunosuppressive agents [6], enzymes [7, 8] and especially antibiotics [9]. Till date, more than 10,000 antibiotics have been isolated from actinomycetes [10]. A few decades ago, this identification of such huge numbers of antimicrobial has led to a misconception that the discovery of bioactive metabolites from actinomycetes might reach its extreme limit. But with the advent of Next Generation sequencing [11] and sophisticated bioinformatics tools, number of whole genome sequences of model actinomycetes [12, 13] became available and analysis of those genome sequences revealed identification of number of rare actinomycetes. Further characterization of these new isolates has lead to isolation and identification of novel bioactive compounds with significant therapeutic potential. According to a mathematical modeling, only 3 % of all antibacterial agents synthesized by *streptomycetes* have been reported so far [14]. In concomitant with this information and emerging problem of multi drug resistance (MDR) and new phyto-pathogens, actinomycetes regained its position as the center of interest and isolation of actinomycetes from relatively unexplored region is again popularized [15, 16]. Recent worldwide exploration especially in China and Japan proved the notion completely relevant [17–19]. India with its enormous geographical diversity offers entirely unique environmental dynamics that support prospering of diverse group of microorganisms which also include different kinds of actinomycetes. Actinomycetes diversity and their biological activities are also a prospective field of research in the Indian context as several new species of this phylum has been reported from this part of the world [20] and significant number of antibiotics patents were registered in recent years [21].

Several research groups of China [22–24] have isolated diverse mangrove actinomycetes and identified them as promising and productive sources of novel bioactive compounds. New actinobacterial compound from Sundarbans has also been reported [25] earlier, which is now compounded with our study. In the present study, actinomycetes were isolated from relatively unexplored regions of Sundarbans. An array of selective isolation and characterization procedures were used to recover and identify diverse actinomycetes from soils prior to establishing their activities. Representative isolates were found to be active against various test organisms including phytopathogens. They were characterized using conventional, morphological and molecular systematic methods as well as on the basis of their ability to utilize different carbon source and chemical compounds. Conditions for the production of metabolite with antimicrobial potential were optimized for the selected strains as to achieve the best yield of secondary metabolite at an accelerating rate [26]. Finally the crude extract from the most active strain SMS-SU21 was analyzed by GC-MS to estimate the actual bioactive potential and the particular bioactive compound responsible for the antimicrobial activity was purified.

## Methods

### Sampling locations for the isolation of actinomycetes

Sediment samples were collected from three different sampling stations, selected on the basis of variation in the anthropogenic influences as well as the salinity gradient of the river Matla, flowing close to the sampling stations (Table 1). To avoid recurrence of identical actinomycetes, five sampling points were set in each sampling site within an area of 400 meter^2^ and then the soil samples were collected at different depths (surface, 7.5 cm, 15 cm, 22.5 cm, and 30 cm) and mixed to generate a composite soil for further analysis. Soil samples (approx. 500 g) were collected using clean, dry, sterilized polythene bags along with hand core and sterilized gloves to avoid any kind of contamination so that the points of collection had as widely varying characteristics as possible with regard to the organic matter, moisture content, particle size and color of soil. Samples were preserved at 4 °C until pretreatment which was required for inhibiting or eliminating unwanted microorganisms. Longitude and latitude of the sample collection points was noted with a GPS tracking device (Trimble juno SB). Physicochemical parameters of the soil samples were measured according to Basak et al. [27].

**Table 1:** Physicochemical parameters and sample site descriptions

Sampling site information	Gadkhali (Station A)	Bonnie camp (Station B)	Kalash (Station C)
Latitude (N)	22°06′32.57″	21°51′05.823″	22°00′25.599″
Longitude (E)	88°46′22.22″	88°38′27.021″	88°42′13.948″
Site description	Under high anthropogenic pressure, i.e., oil leakage, agricultural wastes, commercial market	Isolated island with several small creeks and Inhabited by broad range of mangroves	Located near to the preserved area, under nearly pristine condition
Total isolated actinomycetes	Twenty five	Twenty three	Six
Actinomycetes having broad spectrum antimicrobial activity	Two	Six	One
	(SMS_B,SMS_9)	(SMS_5,SMS_10,SMS_13, SMS_SU13,SMS_SU21,SMS_SU23)	(SMS_7)
Physicochemical parameters			
pH	7.2	7.9	7.7
Salinity (PSU)	14-16	20-22	27-29
Temperature (°C)	31.3	30.5	30.8
Total organic carbon (TOC) (%)	3.56	3.04	3.29
Total nitrogen (N) (%)	1.25	1.8	2.3
C/N ratio	2.8:1	1.6:1	1.4:1

*PSU* practical salinity unit

### Pretreatment of the soil and isolation of actinomycetes

The samples were dried in a laminar flow-hood for a certain period. Once dried, the samples were sieved to exclude large mineral and organic particles followed by selective pre-treatments of the soil samples including heating at 70 °C for 20 min [28], Rehydration, moist incubation and centrifugation [29], treatments with Chloramine-T [30]; phenol (1.5 %, 30 min at 30 °C) [31]; 0.05 % SDS and 6 % yeast extract (40 °C, 200 rpm, 30 min), Calcium carbonate and chitin treatment [32].

The pre-treated soil samples were then dissolved in 0.9 % saline water and serially diluted down to 10^–6^ .100 μl aliquots from solutions were plated on seven selective media supplemented with different concentration of salt up to 18 % (Additional file 1: Table S1). The basal composition of those media as follows, (1) M2 (sodium caseinate-2 g; L-asparagine-0.1 g; sodium propionate-4 g; K_2_HPO-.5 g; MgSO_4,_ 7H_2_O-.1 g; FeSO_4_, 7H_2_0-.001 g; agar-15 g; pH 8.1 ± 0.2), (2) ISP_4_ (starch-8 g; Glycerol-5 ml v/v; K_2_HPO-1 g; MgSO_4,_7H_2_O-1 g; NaCl-1 g; (NH_4_)_2_SO_4_-2 g; CaCO_3_-2 g; FeSO_4_,7H_2_0-1 g; MnCl_2_-1 g; ZnSO_4_-1 g; agar-20 g pH 7.0 ± 0.2), (3) ISP2 (Yeast extract-4 g; malt extract-10 g; Dextrose-4 g;agar-20 g;pH 7.2 ± 0.2), (4) GYM (Yeast Extract-4 g; malt extract-4 g; Glycerol-5 ml v/v; agar-15 g; pH 7.0-7.2), (5) Starch-Casein media (starch-10 g; casein-1 g; KNO_3_- 2 g; KH_2_PO_4_-2 g; NaCl-2 g; MgSO_4,_ 7H_2_O-0.5 g; CaCO_3_-0.02 g; FeSO_4_,7H_2_0-0.001 g; agar-18 g; pH 7.0 ~ 7.4), (6) IM8 [33] (glucose-10 g; peptone-5 g; tryptone-3 g; NaCl-5 g; agar-15 g; pH 7.0,) and (7) cross streak media [CSM] (yeast extract-3 g; peptone-3 g; casein-3 g; starch-8 g; K_2_HPO-0.5 g; MgSO_4,_7H_2_O-0.5 g; NaCl-2 g; agar, 15 g;7.0-7.6). All the above mentioned compositions are for one Liter of media. The isolation media were supplemented with filter sterilized Nalidixic acid & Amphotericin b (50 μg ml^−1^) to inhibit the growth of gram negative bacteria and fungus. All the plates were incubated at 28 °C for 7 to 31 days to target slow growing actinomycetes.

Initially dry, powdery, fuzzy, branched filamentous and aerial filamentous colonies were identified as actinomycetes and they were sub-cultured in M2 media for further characterization

### Morphological identification

Selected actinobacterial isolates were preliminarily subjected to phase contrast microscopy with Zeiss Scope-A1 microscope to observe the branched aerial hyphae arrangements. Spore chain ornamentation and spore surface of the isolates were observed under scanning electron microscope (Zeiss EVO18) with thick gold coating in Ruorum 150 TES for 10 min.

### Biochemical characterization on the basis of carbon source utilization

The GEN III MicroPlate™ test (Biolog Inc., Hayward, CA, USA) was carried out for the selected strains, which provides a standardized micro-method using 94 biochemical tests to profile and characterize a broad range of bacteria [34]. Biolog GEN III MicroPlate analyzes a microorganism depending on phenotypic tests which includes 71 carbon source utilization assays and 23 chemical sensitivity assays. To represent the relatedness between the nine isolates on the basis of Biolog assay, a dendrogram was constructed [35]. Presence of a positive reaction was assigned the binary value 1, and negative reaction was assigned a binary value of 0. For each of the biochemical characteristics, at least one strain differed in any one characteristic. The dendrogram based on Euclidean distances was generated through the hierarchical cluster analysis algorithm using SPSS for Windows Version 20.0 (SPSS Inc. Chicago, IL, USA).

Along with the Biolog test, various classical biochemical tests including starch hydrolysis, catalase, oxidase etc. were also performed using standard protocols [36] for phenotypic fingerprinting and genus level confirmation.

### DNA amplification, sequencing and phylogenetic analysis

Representative actinomycetes strains were inoculated into 250 ml conical flask containing 50 ml GYM medium and cultured at 28 °C with shaking at 150 rpm for 7 days. Genomic DNA was extracted with HiPurA streptomycetes DNA isolation and purification kit (Himedia, India). Concentrations of all the extracted DNA samples were adjusted to 30 ng μl^−1^ for PCR reaction. Universal primers such as Eubac27F and Eubac1492R [37] were not successful for all the strains for amplification of 16S rDNA. To encounter this problem a new forward primer AB1 (5′AGTGGCGAACGGGTG3′) was designed at the second conserved region of 16S rRNA gene from a multiple sequence alignment of available Actinomycetales 16S rDNA gene sequences in the GenBank and used in combination with universal reverse primer 1378R [38] (5′CGGTGTACAAGGCCCGG3′). The PCR reaction was performed in a final volume of 25 μl, which consisted of template DNA 2 μl: molecular grade H_2_O: 15 μl, buffer: 3.5 μl including 12.5 mM MgCl_2_, 1 μl dNTP mix (10 mM each nucleotide),1 μl of forward and reverse primer each (concentration 10 picomole), 0.5 μl (2U) of *Taq*-polymerase and 1 μl DMSO under the following cycling conditions: initial denaturation at 95 °C for 5 min, followed by 30 cycles of 95 °C for 60 s, annealing at 54 °C for 50 s,72 °C for 120 s and final extension of 72 °C for 10 min. PCR products were purified with QiagenQIAquick PCR cleanup kit. PCR amplified templates were sequenced using ABI 3100 Genetic Analyzer (AppliedBiosystems). Sequence data were compiled with the BioEdit program (http://www.mbio.ncsu.edu/BioEdit/bioedit.html) and examined for sequence homology with the archived 16S rDNA sequences from GenBank at http:// www.ncbi.nlm.nih.gov/nucleotide, employing the BLAST search program [39] and EzTaxon server (www.ezbiocloud.net/eztaxon). Multiple sequences were aligned with CLUSTAL W [40]. Phylogenetic analyses were performed according to the neighbor joining (NJ), maximum parsimony (MP) and maximum likelihood (ML) methods using MEGA version 6.0 [41]. To determine the support for each clade, bootstrap analysis was performed with 1000 re-samplings. Sequences were deposited in GenBank (http://www.ncbi.nlm.nih.gov/genbank/index.html) under the accession numbers [GenBank: KJ777668-KJ777676].

### Chemotaxonomic identification

Fatty Acid Methyl Ester (FAME) Analysis was performed for chemotaxonomic identification of the nine isolates. FAME analysis of the isolates was outsourced to the Microbial Type Culture Collection and Gene Bank (MTCC), Institute of Microbial Technology, Chandigarh, India. Analysis was done using standard gas chromatography and applying then Sherlock MIS system.

### Bacterial and fungal test organism used for this study

Bacterial strains such as *Escherichia coli* (ATCC 25922), *Escherichia coli* (ATCC 8739) *Staphylococcus aureus* (ATCC 25923), *Bacillus subtilis* (ATCC6633), *Vibrio cholera* (MTCC 3906), *Pseudomonas aeruginosa* (ATCC 27853), *Enterobacter aurogenesa* (ATCC 13048), *Salmonella typhi* (ATCC 6539), *Salmonella typhimurium* (ATCC 14028) and fungal strains *Saccharomyces cerevisiae* (ATCC 9763), *Candida albicans* (ATCC 0231), *Aspergillus niger* (ATCC 16404) were used as test organisms for the study of antimicrobial potential of the isolated strains. The selected actinobacterial strains were also tested against plant pathogens such as *Ustilago maydis* SG200, *Rhizoctonia solani* AG1-1A and *Macrophomina phaseolina* R9.

### Preliminary screening for antibacterial activity

The antimicrobial activity of all the 54 pure isolates were qualitatively determined by perpendicular streak method [42] on cross streak media as this media supports the growth of actinobacteria, as well as bacteria and fungi which were used as test organisms in this study.

### Extraction of secondary metabolites and preparation of crude extract

The following three strains (SMS_SU21, SMS_SU13 and SMS_7) with the best inhibitory activity were cultivated in 2.5 liter of modified cross streak media [yeast extract: 3 g; peptone: 3 g; casein: 3 g; starch: 8 g; glycerol: 3 g; CaCO_3:_ 0.75 g; K_2_HPO: 0.5 g; MgSO_4,_7H_2_O: 0.5 g; NaCl: 12 g, pH 7.4] at 28 °C with a shaking speed of 150 rpm during an incubation period of 8 days for SMS_SU13 and SMS_SU21 whereas 12 days for SMS_7. After incubation all the cultures were centrifuged at 6000 × G (Sorvall Legend ×1R, Thermo Scientific, rotor: F15-6X-100Y) for 15 mins and supernatants were collected, filtered with 0.45 μmWhatman filter paper and subsequently extracted with ethyl acetate (1:1) twice. Residual ethyl acetate was evaporated with rotary evaporator (Eyela, Tokyo, Japan) at 40 °C temperature. Finally the extracts were dissolved in HPLC grade methanol to maintain a concentration of 50 mg ml^−1^approximately.

### Quantitative assay for antimicrobial activity

The crude extracts of SMS_SU21, SMS_SU13 and SMS_7 were used for quantitative assay by agar well diffusion technique [43]. 100 μl of freshly prepared bacterial test organisms were spread on Mueller Hinton agar media whereas fungal test organisms were spread on Potato dextrose agar (PDA). 50 μl of crude extract was used in each of the well. Methanol used as a control. Plates were incubated at 37 °C for 24 hour (for bacteria) and at 28 °C for 72 hour (for fungus). The level of growth inhibition was assessed by the average zone of inhibition diameter recorded (4 replicates). ‘++++’ = zone of inhibition ≥ 25 mm; ‘+++’ = zone of inhibition ≥ 20–24 mm; ‘++’ = zone of inhibition ≥ 10–19 mm; ‘+’ = zone of inhibition ≥ 5–9 mm; and ‘-’ = no zone of inhibition. [Well diameter (5 mm) was included].

### Determination of Minimum Inhibitory Concentration (MIC)

The minimum inhibitory concentrations (MIC) of the crude extracts were determined against all the test organisms using well diffusion method. Initial concentration of the crude extract was 50 mg ml^−1^, which was then diluted to four different concentrations of 5 mg ml^−1^, 0.5 mg ml^−1^, 0.05 mg ml^−1^, 0.005 mg ml^−1^ and used to evaluate the efficiency of antimicrobial attributes. MIC was defined as the lowest concentration that produced zone of inhibition ≥5 mm in diameter against test organisms [44].

### Antioxidant properties

#### DPPH radical scavenging assay

DPPH quenching ability of the crude extracts of SMS_SU21, SMS_SU13, and SMS_7 was measured according to [45]. A methanol DPPH solution (0.15 %) was mixed with serial dilutions (0.0625, 0.125, 0.25, 0.5,1 mg ml^−1^) of the crude extracts and after 10 min, the absorbance was read at 515 nm. The radical scavenging activity was expressed as IC50 (mg ml^−1^), (the dose required to cause a 50 % inhibition). Vitamin C was used as standard. The ability to scavenge the DPPH radical was calculated by the following:

#### Formula

DPPH radical scavenging activity (%)

1 $$ = \left({\mathrm{A}}_0\hbox{-} {\mathrm{A}}_1\right)\ /\ {\mathrm{A}}_0\times 100 $$

Where A_0_ is the absorbance of the control at 30 min and A_1_ is the absorbance of the sample at 30 min. All samples were analyzed in triplicate_._

### Condition optimization for growth as well as bioactive compound production

#### Media optimization

Four different media namely M2, CSM, IM8 and ISP4 were inoculated with 50 ml of culture in 250 ml conical flasks and incubated at 150 rpm for 8–12 days at 28 °C. After incubation the culture was centrifuged at 6000 × G (Sorvall Legend X1R, Thermo Scientific, rotor: F15-6×-100Y) for 15 mins, the biomass was dried for 24 hours at 60^0^c and the dry weight of the mycelium was measured & correlated with the growth of the isolates. The supernatant was extracted with ethyl-acetate (1:1) and finally dissolved in 250 μl of methanol to evaluate the antimicrobial effects by well-diffusion method against previously mentioned test organisms. All experiments were performed thrice in duplicate sets.

### Growth parameter optimization

*Effect of Incubation Periods*: M2 broth (for SMS_7) and CSM broth (for SMS_SU13 and SMS_SU21) were inoculated and incubated for 24, 96, 192 and 288 hours. After each time point, the extracts were prepared and tested for antimicrobial activities. The mycelial dry weights of the strains were also measured.

*Effect of Temperature*: The strains were inoculated into suitable media (as mentioned above) and incubated at different temperatures viz. 16 ± 2 °C, 22 ± 2 °C, 28 ± 2 °C and 34 ± 2 °C for 7 days. After incubation, the extracts were prepared and tested for antimicrobial activities. The dry weights were also measured in each case.

*Effect of salinity*: selected media (as mentioned above) were prepared with different salinity concentrations (3 %, 6 %, 9 % and 12 %) by adding NaCl. The strains were inoculated and incubated at 28 ± 2 °C for 7 days. After incubation, the extracts were prepared and tested for antimicrobial activities. The dry weights were also measured in each case.

All the experiments were performed in triplicates and mean values along with the standard deviations have been represented here.

### Purification of the bioactive compound from SMS_SU21

The crude extract was subjected for silica gel thin layer chromatography (TLC) with a solvent system of methanol: chloroform (2:8). The TLC fractions were extracted from the scraped spots and checked for antimicrobial activity against various test organisms including plant pathogens. Finally the active principal component of SMS_SU21 was separated by HPLC (Waters515) instrument with a Nova-pak HR-C18 column (7.8X300 mm). Mobile phase was a gradient of water:acetonitrile (90:10 for 0–5 min, 40:60 for 5–15 min, 10:90 for 15–23 min, and 5:95 for 23–30 min), flow rate 1.0 ml min^−1^, UV detection at 275 nm with Waters™486 tuneable absorbance detector. The fraction with bioactive principal was pooled and dissolved in 100 μl methanol to achieve 50 μg ml^−1^ concentrations.

### GC-MS analysis of SMS_SU21

Identification of the chemical compounds present in the crude extract was carried out by GC-MS. Analysis was conducted on a Factor four™ capillary column (VF-5 ms, 30 m, 0.25 mm id, 0.25 μm film thickness; Varian, Middelburg, The Netherlands) with the following conditions: constant flow of Helium, 1.0 ml min^−1^; the inlet temperature 285 °C remain the fixed throughout the analysis; injection volume, 2 μl (LVI) in the liner with an open purge valve (30:1 split ratio) initially and closed at 0.00 min, and open again (30:1) at 26.00 min and remain open till the end of the run; oven temperature program, 80 °C for 2 min, then 18 °C min^−1^ ramp to 260 °C and held for 6 min, again 4 °C min^−1^ ramp to 285 °C and held for 6 min. The MS instrument transfer line temperature was 280 °C, with 220 °C ion trap and 120 °C manifold temperatures. Full-scan (40–650 m/z) EI (auto) mode with20 μA filaments current was used for MS analysis from 5.00–28.00 min, which gave 0.78 s/scans (3 μ scan). Target automatic gain control was 20,000, and the multiplier voltage was 1450 V. Baseline offset −5, peak find with S/N of the quantifier ion at least 3 and peak width 2 s was set as the parameters for processing the peaks in the chromatograms. Minimum similarity match with regards to the NIST library spectra was kept at 500 (reversed fit). Quantification was done on the basis of diagnostic ion and the peak assignments and integration were automatically done through software.

## Results

A total of 54 actinomycetes were isolated at different locations in Sundarbans, among which 25 (46.29 %) from Gadkhali, 23 (42.59 %) from Bonnie camp and 6 (11.11 %) from Kalash (Table 1). All the isolates survived through various soils pre-treatment programmes which suggest them to be genetically well-equipped and able to maneuver various survival strategies even in a competitive environment like Sundarbans mangrove. All the isolates were subjected to primary screening for antimicrobial activity where six strains (SMS_5, SMS_10, SMS_13, SMS_SU13, SMS_SU21, SMS_SU23) from Bonnie camp were found to possess antimicrobial activity and only two strains (SMS_B, SMS_9) from Gadkhali and only one strain (SMS_7) from Kalashwere active against various test organisms. These nine bioactive strains were further selected for poly-phasic identification.

### Identification of actinomycetes

#### Morphological, biochemical and chemotaxonomical analysis

**Fig. 1:**
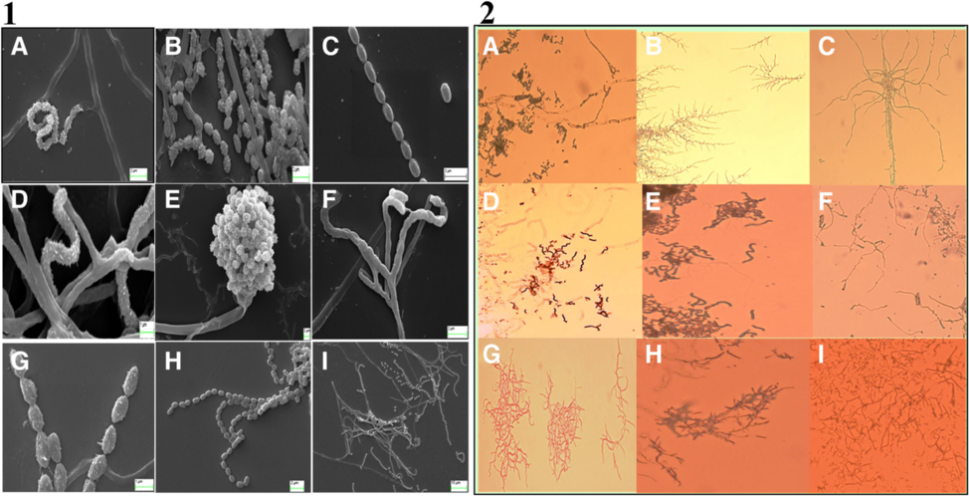
Panel **1**- Morphology of sporophores of nine Actinomycetes studied by scanning electron microscopy [(*a*) SMS_B, (*b*) SMS_5, (*c*) SMS_7, (*d*) SMS_9, (*e*) SMS_10, (*f*) SMS_13, (*g*) SMS_SU13, (*h*) SMS_SU21, (*i*) SMS_SU23]. Scale bar shown on each photomicrograph. Panel **2**- Arrangement of mycelium studied by phase contrast microscopy. [Phase contrast micrographs of nine actinomycets (*a*) SMS_B (*b*) SMS_5 (*c*) SMS_7 (*d*) SMS_9 (*e*) SMS_10 (*f*) SMS_13 (*g*) SMS_SU13 (*h*) SMS_SU21 (*i*) SMS_SU23 . Magnification 40 × .]

Seven selective media (Additional file 1: Figure S1 and Additional file 1: Table S3) were used to observe the distinct colony morphology of the isolates. The colours of the aerial and substrate mycelium were described according to the colours of the RALcode. Aerial hyphae arrangements, Spore chain ornamentation and spore surface of the isolates were subsequently observed by phase contrast and scanning electron microscopy (Fig. 1), indicating variation among the sporophore sizes and ornamentation and even in the spore surface, especially SMS_10 possessing a unique bouquet like spore arrangement.

Classical biochemical test evidenced that all the isolates were positive in Starch Hydrolysis, Casein Hydrolysis, and Catalase Test and negative for oxidase test. Results of other biochemical tests have been summarized in (Additional file 1: Table S2).

**Fig. 2:**
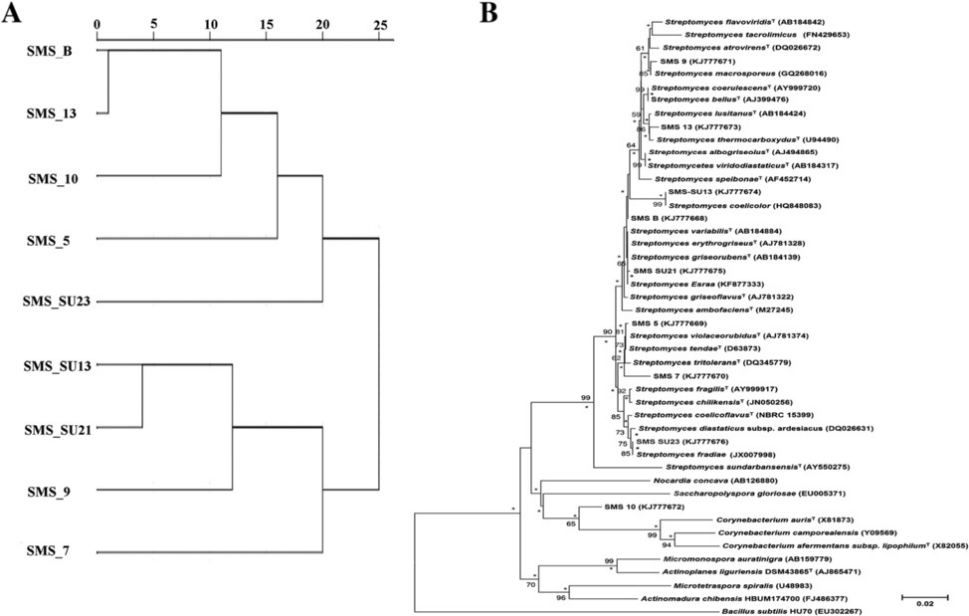
Panel **a**-Dendrogram based on Euclidean distances of 71 carbon sources and 23 chemical utilization characteristics for nine actinomycetes. This dendrogram was constructed by using average linkage (between groups). Panel **b** - Phylogenetic tree based on 16S rRNA gene sequences obtained by the neighbor-joining (NJ) method showing the phylogenetic relationships among nine actinobacterial phylotypes of this study (shown in boldface) and related actinobacteria. Numbers at nodes indicate levels of bootstrap support (%) based on a NJ analysis of 1,000 resampled datasets, only values greater than 50 % are shown. Asterisks indicate branches that were also recovered using the maximum parsimony (MP) and maximum likelihood (ML) algorithms. NCBI accession numbers are given in parentheses. Bar 0.02 nucleotide substitutions per site. The sequence of *Bacillus subtilis* HU70 (EU302267) was used as the out-group

The dendrogram (Fig. 2a) of the nine isolates was constructed based on the Biolog assay indicating species relatedness (Additional file 1: Table S4). The dendrogram differentiated the strains into two broad groups. Strains SMS_7, SMS_9, SMS_SU21, SMS_SU13 formed the first group while SMS_5, SMS_B, SMS_10, SMS_13, SMS_SU23 constituted the second group.

The result of the fatty acid methyl ester (FAME) analysis is shown in Additional file 1: Table S5. The predominant cellular fatty acids of the nine actinomycets isolates were 14:0 ISO (3.63 %-7.40 %), 15:0 iso (7.64 %-15.64 %), 15:0 anteiso (14.18 %-23.58 %), 16:0 ISO (16.45 %-33.13 %),16:0 (5.16 %-15.76 %),17:0 anteiso (6.25 %-11.04 %) and 17:0 iso (2.19 %-9.30 %) which closely matched those of streptomycetes but considerable differences were recorded among the nine isolates.

#### Molecular phylogenetic analysis

Direct PCR amplification of 16S rRNA gene from a single isolate demonstrated excellent congruence between morphology and 16S rRNA sequence data. A phylogenetic comparison (Fig. 2b) of these sequences with the databases of valid species by using NCBI and EzTaxon server revealed that eight isolates were representing the genus *Streptomyces* whereas SMS_10 showed only 93.57 % sequence similarity with *Streptomyces albogriseolus* NRRL B-1305^T^ and 92.5 % sequence similarity with *Corynebacterium auris* DSM 328^T^. When compared with available type strains only (Additional file 1: Table S6), it was found that among the eight isolated strains SMS_SU21 showed 99.75 % similarity with *Streptomyces griseorubens* NBRC 12780^T^and shared a close phylogenetic relationship with SMS_B which is 98.62 % similar to *Streptomyces variabilis* NBRC 12825^T^. According to EzTaxonSMS_SU13 shared only 96.59 % sequence similarity to the type strains like *Streptomyces labedae* NBRC 15864^T^, *Streptomyces variabilis* NBRC 12825^T^ and *Streptomyces erythrogriseus* LMG 19406^T^. SMS_9 is 98.21 % similar to *Streptomyces atrovirens* NRRL B-16357^T^,SMS_SU23 is close to *Streptomyces coelicoflavus* NBRC 15399^T^ whereas SMS_13 is almost 99 % similar to *Streptomyces lusitanus* NBRC 13464^T^. Both SMS_5 and SMS_7 are vary closed to *Streptomyces tendae* ATCC 19812^T^ but they are quite district from each other in respect to other characteristics.

### Antimicrobial activity

Initially all the 54 pure isolated actinomycetes strains were subjected to preliminary antimicrobial assay by perpendicular streak method. Only nine out of the fifty four isolates showed antimicrobial activity against both bacterial and fungal test organisms. These nine isolates were selected for characterization studies. Depending on higher degree and broader range of antimicrobial production (actinobacterial strains which are active against almost all the test organisms), only three strains were selected out of the nine strains for further bioactivity studies. Antimicrobial activity was assessed with the crude extracts of SMS_SU21, SMS_SU13 and SMS_7 (Table 2). Noticeable development was recorded when; SMS_SU21 inhibited almost all the bacterial as well as fungal test organisms and even the plant pathogenic fungal strains under study to a greater extent. Maximum activity was recorded against *Vibrio cholerae* (34 ± 2 mm) and *Staphylococcus aureus* (30 ± 1 mm) with a MIC value of 0.05 mg ml^−1^. On the other hand SMS_SU13 proved to be more effective against Gram negative organisms, especially against *Pseudomonas aeruginosa* (30 ± 2 mm) with a MIC value of 0.05 mg ml^−1^ than Gram positive and fungal test organisms. SMS_7 is moderately active against bacterial test organisms (MIC value ranging between 0.5-5 mg ml^−1^) though better activity was recorded against all fungal strains (MIC value ranging between 0.05-0.5 mg ml^−1^).

**Table 2:** Antibacterial and Antifungal activity of crude ethyl acetate extracts of SMS_7, SMS_SU13 and SMS_SU21

Test organism	SMS_7		SMS_SU13		SMS_SU21	
	Zone of inhibition (mm)	MIC (mg ml^−1^)	Zone of inhibition (mm)	MIC (mg ml^−1^)	Zone of inhibition (mm)	MIC (mg ml^−1^)
*Escherichia coli* (ATCC 25922)	++	5	+++	5	+++	0.5
*Escherichia coli* (ATCC 8739)	++	5	++	0.5	++	0.05
*Staphylococcus aureus* (ATCC 25923)	++	0.5	+	5	++++	0.05
*Bacillus subtilis* (ATCC 6633)	++	0.5	+	5	+++	0.5
*Pseudomonas aeruginosa* (ATCC 27853)	+	0.5	++++	0.05	+++	0.05
*Enterobacter aerogenes* (ATCC 13048)	++	5	++	5	+++	0.5
*Salmonella typhi* (ATCC 6539)	+	5	+++	0.5	+++	0.5
*Salmonella Typhimurium* (ATCC 14028)	+	5	+++	0.5	++	0.5
*Vibrio cholerae* (MTCC 3906)	+++	0.5	+++	0.5	++++	0.05
*Saccharomyces cerevisiae* (ATCC 9763)	+++	0.05	-	-	+	5
*Candida albicans* (ATCC 10231)	++	0.5	+	5	++	0.5
*Aspergillus niger* (ATCC 16404)	+++	0.05	++	5	+++	0.5
*Ustilago maydis SG200*	+++	0.05	++	5	++	0.5
*Macrophomina phaseolina R9*	+++	0.5	+	5	++	5
*Rhizoctonia soloni* AG1-1A	++	0.5	+	5	++	0.5

The level of growth inhibition was assessed by the average zone of inhibition diameter recorded (4 replicates). ‘++++’ = zone of inhibition ≥ 25 mm; ‘+++’ = zone of inhibition ≥ 20–24 mm; ‘++’ = zone of inhibition ≥ 10–19 mm; ‘+’ = zone of inhibition ≥ 5–9 mm; and ‘-’ = no zone of inhibition. [Well diameter (5 mm) was included]. MIC was defined as the lowest concentration that produced zone of inhibition ≥5 mm in diameter against test organism

### Antioxidant activity

#### DPPH radical scavenging activity

**Fig. 3:**
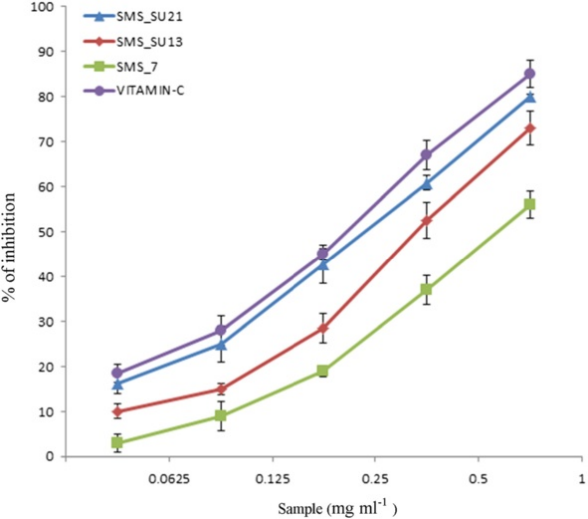
Antioxidant activity: − DPPH radical scavenging activity of the crude extracts of SMS_SU21, SMS_SU13 and SMS_7 at different concentrations (0.0625-1 mg ml^−1^). Vitamin C was taken as positive control. Each value presents the mean ± SEM of triplicate experiments

Figure 3 shows that SMS_SU21 possesses strong antioxidant property and it’s IC50 value (0.242 ± 0.33 mg ml^−1^) is very close to the IC50 of vitamin (0.225 ± 1 mg ml^−1^) which is a potent antioxidant. SMS_SU13 with an IC50 of 0.356 ± 0.66 mg ml^−1^ can be considered as moderate antioxidant producer whereas SMS_7 (IC50 0.609 ± 2 mg ml^−1^) showed weakest antioxidant property among this three actinomycetes.

#### Condition optimization for growth and bioactive compound production

**Fig. 4:**
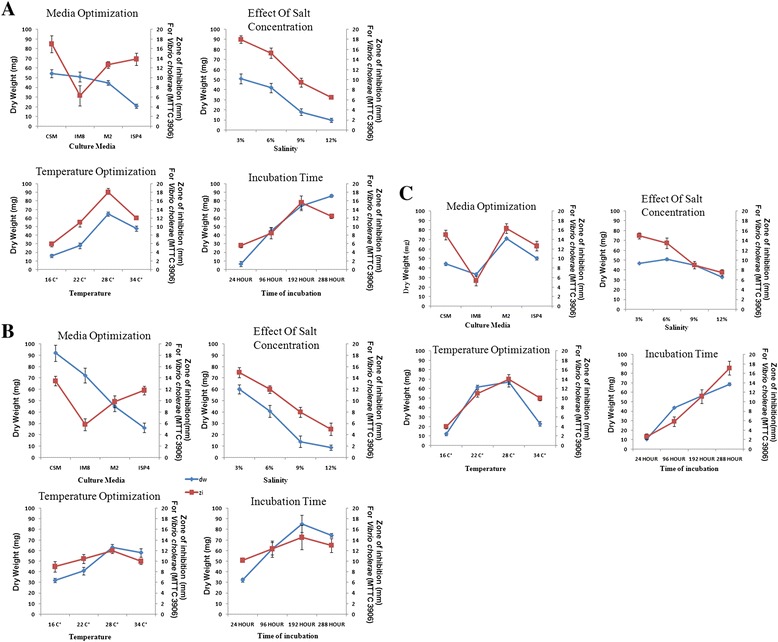
Condition optimization for the strains: **a** SMS_SU21, **b** SMS_SU13, **c** SMS_7. Dry weight was correlated with growth and antimicrobial activity was measured against *Vibrio cholera* (MTCC 3906)*.* [dw-dry weight, zi-zone of inhibition]

Media optimization is a crucial factor for production of bioactive compounds, as proper nutritional composition only leads to the expression of actual genes and triggers the required metabolic pathways [46]. Here we found that cross streak media was the most favorable one for growth as well as for the production of bioactive compound for SMS_SU21 and SMS_SU13 whereas SMS_7 preferred M2 medium for growth and activity (Fig. 4). ISP4 with its inorganic nitrogen sources possibly triggers a stress condition. Though this stress is not suitable for biomass production but is very useful for secondary metabolite production for all the tested isolates (Fig. 4). Exactly contradictory result was observed in case of IM8 media where glucose was used as sole carbon source. This media supported cell mass production for most of the strains but certain decrease of activity was observed for all the strains.

Actinomycetes are known for their slow growth, therefore little noticeable increase in biomass production was observed until 96 hours of incubation was reached for all the isolates. Generally actinomycetes produce secondary metabolites after attainment of stationary phase, which was achieved by SMS_SU13 and SMS_SU21 after 192 hours whereas SMS_7 took much longer time (288 hours) to produce bioactive secondary metabolites. Efficiency of antimicrobial activity reduced considerably after a certain incubation time. It may be because of the degradation of active compound.

Growth and antimicrobial activity were observed in the temperature range 16 °C-34 °C. In this study, all the three bioactive strains achieved their optimum growth and highest bioactivity at 28 °C.

Salinity is a key factor to regulate the ecosystems in Sundarbans mangrove since a salinity gradient was observed from station to station affecting the microbial community profile [47]. We noticed that all the isolates were able to grow and remain bioactive up to a salinity level as high as 12 %. SMS_SU13 and SMS_SU21 showed a gradual decrease in growth and activity from 3 % to 9 % although SMS_7 achieved its highest biomass at 6 % salinity which indicated that SMS_7 was the most halophilic one among the three isolates. At 12 % salinity, growth and activity was severely affected possibly due to extreme osmotic pressure for all the strains but it never ceased entirely.

### Purification and potential fractions extraction from SMS_SU21

HPLC of the crude extract of SMS_SU21 produced five potential fractions which were tested for various biological activities. In respect to antimicrobial activity, fraction SMS_SU21-C which presented a single sharp peak showed desired activity (Additional file 1: Figure S2). This active fraction was subjected to antimicrobial activity assay and enhanced activity against various test organisms was achieved (Additional file 1: Table S7).

### Identification of potential bioactive compounds by GC-MS

**Fig. 5:**
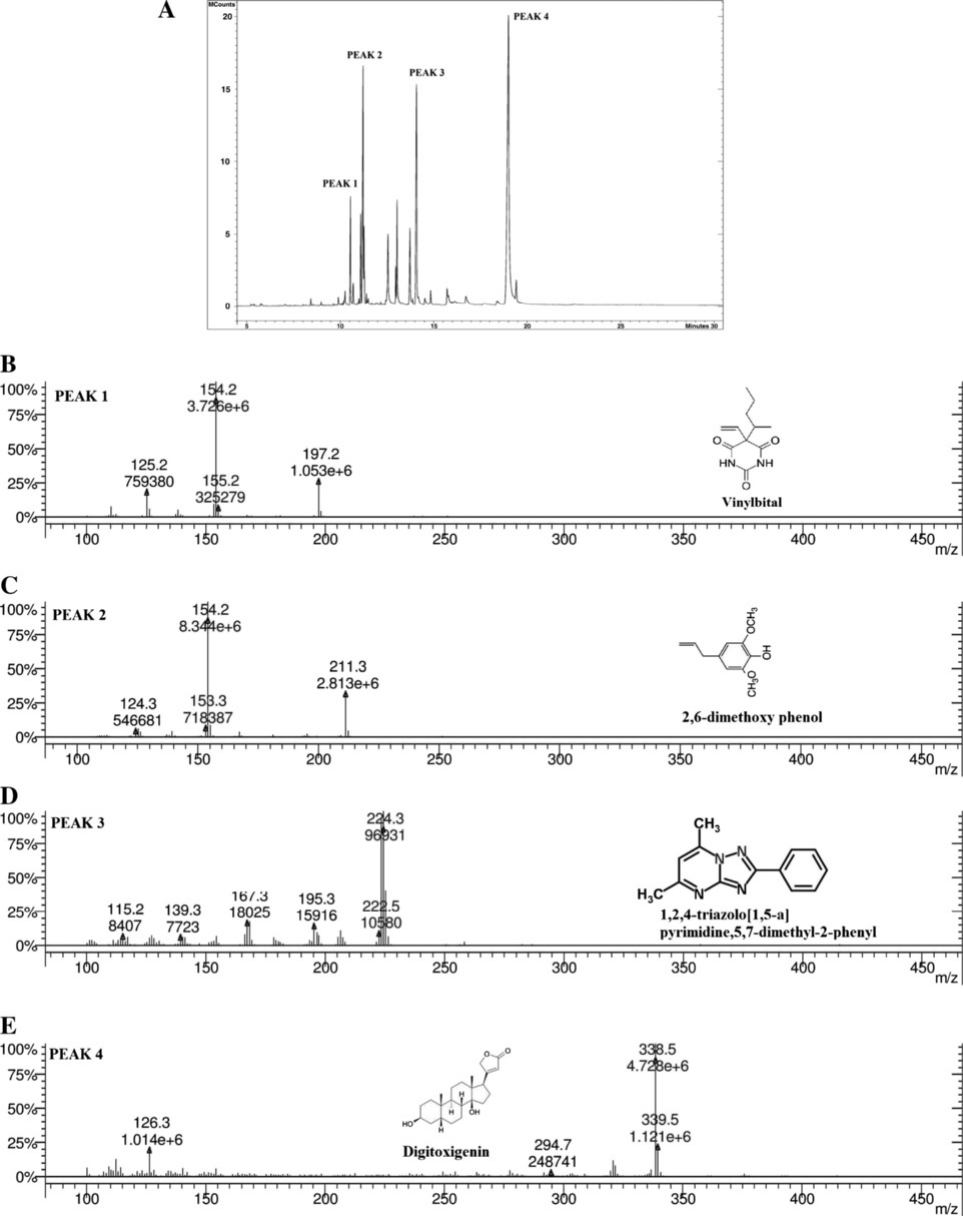
GC-MS chromatograph of SMS_SU21 crude extract with four major peak indicating the presence of bioactive compounds (according to NIST database)

Identification of the volatile components was carried out using GC-MS analysis. The GC-MS chromatogram of the SMS_SU21 crude extract showed a total of 24 peaks (Fig. 5). When compared with NIST database, the nearest compound hits for those peaks were found, the molecular weight and the bioactivity of the compounds corresponding to the 24 peaks are presented in (Additional file 1: Table S8). NCBI PubChem bioassay database (https://pubchem.ncbi.nlm.nih.gov), Dr. Duke’s Phytochemical and Ethnobotanical Databases (http://www.ars-grin.gov/duke) revealed that among the 24 peaks, 16 compounds indicating the presence of bioactive constituents which have been previously reported for their antimicrobial activity [2, 2-propyl-N-ethylpiperidine; 4-Dichloromethyl-5 6-epoxy-2-methoxy-4-methyl-2-cyclohexenone;1,3-cyclopentanedione,2-isopentyl and isoquinoline-1-carbonitrile] that includes Phyto-phathogenic activity [1,2,4-Triazol [1,5-a] pyrimidine,5,7-dimethyl-2-phenyl] too. Other predicted compounds were reported for their pesticidal, antitumor genic, antioxidant, Cardenolidic activities but they were not isolated from actinomycetes earlier.

## Discussion

**Fig. 6:**
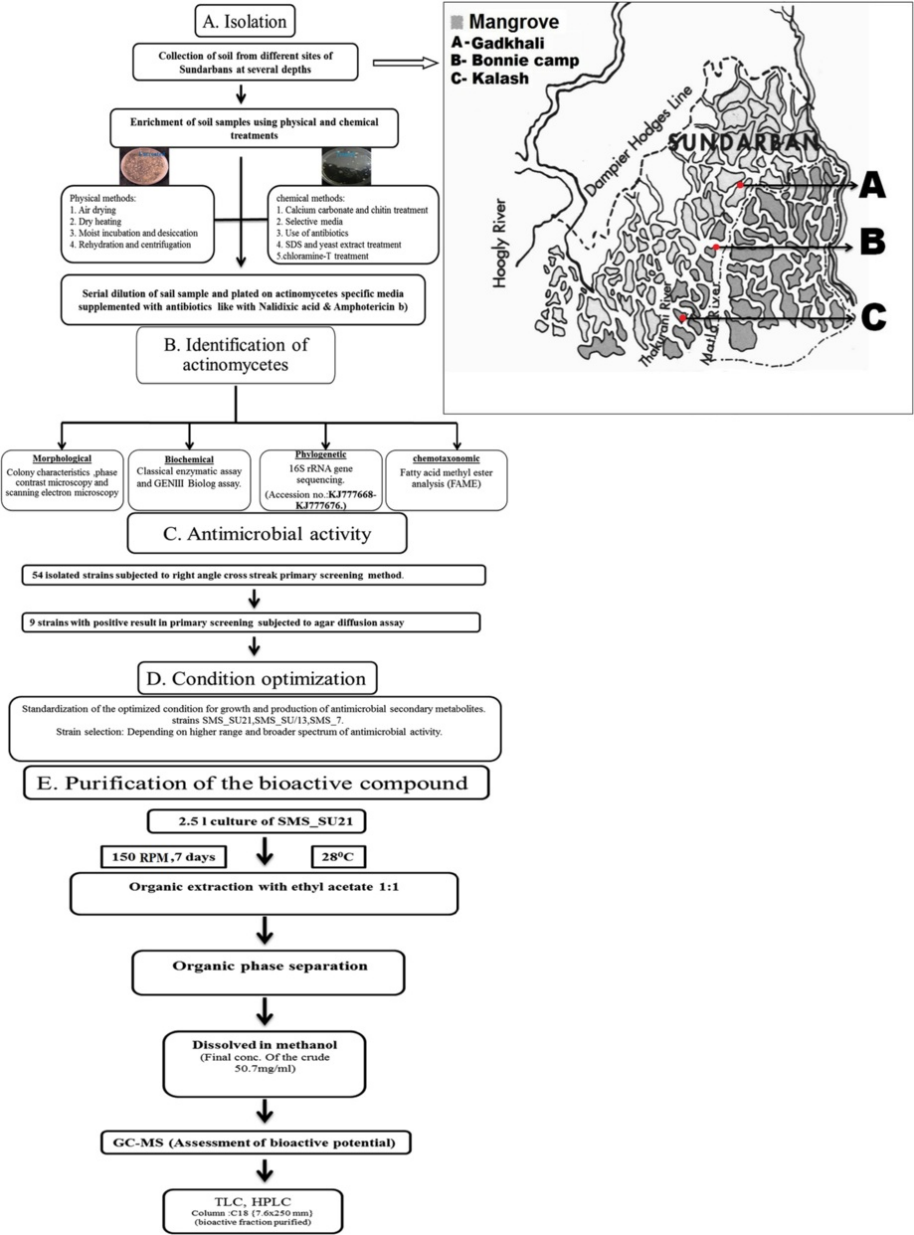
A schematic diagram of the total work plan of the study (Inset: Sampling stations of the area under study)

Sundarbans, the largest delta in the world, located at the mouth Ganga Brahmaputra-Meghna River system with 10,200 km^2^ of Mangrove Forest, extends over two countries namely, India (about 4,200 km^2^) and Bangladesh (6,000 km^2^ approximately). In both the countries, it has been declared as Reserve Forest. Indian Sundarbans lies between 21^0^ 30′ to 22^0^ 15′ North Latitude and 88^0^ to 89^0^ 9′ East Longitude. It consists of 104 islands out of which mangroves are present in 56 islands under 22 forest blocks. The study area of the present work is in the western part of Indian Sundarbans, which is under the administrative control of South 24 Parganas Forest Division and extends in north–south direction. Very few studies of isolating actinomycetes with antimicrobial potential has been reported from this part of the world [48, 49], of which actinomycetes with antagonistic potential were collected mainly from Lothian island [49]. For carrying out this study, soil samples were collected from three locations namely, Gadkhali, Kalash and Bony Camp, which were proved to be enriched source of bioactive actinomycetes (Inset Map in Fig. 6).

Gadkhali is an easily accessible area, about 30 kilometers by road from Sonakhali, a popular business point in Sundarban. It is one of the major entry points in Sundarbans. Being located just opposite to Gosaba Block with well-constructed jetty, it is used as the major navigational point for movement within Sundarbans. A number of ferry services to different islands and points, both passenger service and also cargo, are there starting from this junction. As a result, this area is visited by thousands of people and also hundreds of boats on each day. In our study, though most number of actinomycetes (25) were isolated from Gadkhali but only two (8 %) among them were able to produce compounds with antimicrobial activity. This might be due to the reason that Gadkhali remains under high anthropogenic pressure, i.e., oil leakage, agricultural wastes, and commercial market. In this particular location encountering the mentioned factors would be more important than surviving interspecies competitions for the microorganisms. Quite natural, the diversity of species of microbes available and consequently identified here are comparatively less than those of Kalash and Bonnie camp, which are comparatively undisturbed and pristine in nature. Bonnie camp, an isolated island with several small creeks, inhabited by broad range of mangroves such as sundari (*Heritiera fomes and Heritiera littoralis*), gewa (*Excoecaria agallocha*), goran (*Ceriops decandra*) and keora (*Sonneratia apetala*)) was proved to be the most bioactive site as 6 out of 23 (26.08 %) isolates produced antimicrobial compounds. The probable reason might be the network of creeks, which carries water from all the part of Sundarbans and these intermixing increases the microbial diversity encouraging competition within the microbial population for nutrient and other factors. Another pertinent reason may be the presence of diversified groups of mangroves in Bony camp area, since there are few mangrove species which induces the growth of bioactive Actinomycetes [50]. In contrary, only six actinomycetes were isolated from Kalash which is located near the reserved area, under pristine condition. Among six isolates only one isolate came out to be positive for antimicrobial activity (Table 1). A schematic diagram indicating the screening procedure and characterization in this study is provided in (Fig. 6).

The polyphasic identification of the selected strains revealed that proximity or distancing of different strains in phylogenetic profile (Fig. 2b) is independent of the carbon source utilization profile (Fig. 2a), biochemical characterization (Additional file 1: Table S2),chemotaxonomic analysis (Additional file 1: Table S5) or antimicrobial potential of them. These characters of a strain were observed to be regulated by its habitat, i.e., site of origin. For instance isolates like SMS_5 and SMS_7 are very close phylogenetically (Fig. 2b) but when they were analyzed according to their morphology and antimicrobial potential, significant variation was observed in their characteristics. Difference in natural habitat may be the reason as first one was isolated from Bonnie Camp and the second one from Kalash. This type of anomalous behaviour was also noted in case of SMS_SU13 and SMS_B.

Carbon source and chemical source utilization assay exposed the metabolic edifice of the selected strains revealing metabolic links among them. The dendogram (Fig. 2a) constructed from this assay indicated that SMS_B and SMS_13 in accordance to their relative phylogenetic similarity utilizes similar kind of carbon sources. On the other hand phylogenetically distant strain SMS_10 occurred in that same cluster suggesting a scenario of environmental adaptation due to the accessibility of similar nutrient sources. This cluster also included SMS_5 and SMS_SU23 which utilized majority of available compounds. Other three strains, namely, SMS_SU13, SMS_SU21, and SMS_9 coincide with a single cluster whereas SMS_7 formed an entirely new branch within this cluster because of its pristine natural habitat. Detailed carbon and other chemical utilization information has been provided in Additional file 1: Table S4 which indicates, that, most of the strains preferred polysaccharides over monosaccharides and other derivatives of carbon sources such as D-Galactose, D-Fructose, D-Raffinose or N-Acetyl-β-D-Mannosamine, N-Acetyl-D Galactosamine, 3-Methyl Glucose etc. All the amino acids specially L-histidine and L-Aspartate were utilized at an accelerated rate. Inosine and 1 % Sodium Lactate were utilized by all the strains. Few chemicals that include antibiotics (Troleandomycin, Rifamycin SV, Minocycline, Vancomycin, Lincomycinetc), surfactants (Niaproof4), dyes (Tetrazolium Violet, Tetrazolium Blue) and inhibitor of mammalian cells proliferation (Sodium Butyrate) were also supplied as utilization source. Interestingly a few strains such as SMS_5, SMS_7 and SMS_SU23 indicated positive result for utilization of those compounds which should be marked for prospective industrial application of those strains.

The polyphasic identification of SMS_10 deserves a special mention as in 16SrRNA gene sequencing, it was noted to be only 92.5 % similar to the nearest blast hit *Corynebacterium auris* according to EzTaxon, having a unique sporophore ornamentation that has never been reported earlier (Fig. 1 panel 1*e*). With its unique carbon source utilization pattern and other characteristics it is assumed to be an entirely new genus though further analysis and characterization of this isolate is necessary.

Utilization of different media clearly suggested the effect of catabolic repression [51] as glucose being sole carbon source (IM8) caused significant decline in antimicrobial potential of all the selected isolates. On the other hand starch as a carbon source was noted to be very useful for growth and activity of all the isolates. Ability to fix nitrogen from inorganic salt confirmed the prototrophic nature of the isolates and in the absence of organic nitrogen source (ISP4), isolates grew at a slower rate though antimicrobial activity was not affected (Fig. 4).

Salt tolerance in actinomycetes and their antimicrobial potential was previously reported from India [52, 53] as well as from other part of the world [54]. Most of them were isolated from hyper saline saltpans, and salt lakes. However, very few reports are available regarding Actinomycetes isolated from mangroves [55]. In our study, isolated strains from the mangrove estuarine ecosystem were apparently either borderline extreme halophiles (growing best in media containing 1.5–4.0 M salt) or moderate halophiles (growing best in media containing 0.5–2.5 M salt). According to this scheme, two isolates SMS_SU13 and SMS_SU21 should be categorized under moderate halophiles as they grew best in 0.5–1.7 M salt but SMS_7, isolated from Kalash (isolated under “high salt content medium” pre-treatment program) might be considered as borderline extreme halophile as it can grow up to 15 % (2.5 M) salt concentration (Fig. 4).

The antimicrobial potential and antioxidant property of the three strains has been represented respectively in Table 2 and Fig. 3. After condition optimization each of the strain achieved their appropriate antagonistic potential against the test organisms. Furthermore these three strains showed fungicidal activity against phyto-pathogens such as *Rhizoctonia soloni* AG- 1A which is a necrotrophic fungus, causing rice sheath blight, the most devastating and intractable diseases of rice, *Macrophomina phaseolina* R9 responsible for charcoal rot on many plant species including *Zea mays* and *Pinus elliottii* and *Ustilago maydis* SG200, a potent pathogen for maize. Among the three strains SMS_7 showed 99 % 16 s rDNA sequence similarity to *streptomycetes tendae* which produces nikkomycin [56], well-known peptidyl nucleoside antibiotics mainly effective against fungal pathogens and insects. In this study, SMS_7 was identified to be the most effective strain against fungal strains including phyto pathogens. In addition to antifungal activity, this strain also showed moderate antibacterial activity and low antioxidant property. In16s rDNA sequencing, the strainSMS_SU13 showed 99.5 % similarity to *Streptomyces coelicolor* which is one of the most potent producers of antibiotics like actinorhodin [57]. Along with antimicrobial activity this strain also exhibited moderate antioxidant property which was never reported from any strain *of Streptomyces coelicolor*. SMS_SU21 showed highest degree of antimicrobial activity and antioxidant producing potential among the three isolates . This particular strain showed 99 % similarity to *Streptomycetes griseorubens* and *Streptomycetes esraa* in 16 s rDNA sequencing, which are known for cellulase [58] activity xylanase [59] activity and enzymatic saccharification [60] but search in the Antibiotics Database (http://www.nih.go.jp/∼jun/NADB/search.html) did not evidence any antibiotic production. GC-MS analysis of SMS_SU21 indicated the presence of at least 16 bioactive compounds (Additional file 1: Table S8) which makes this particular strain a very potential one to be more persuasive candidate for further study. Among the noted compounds isoquinoline-1-carbonitrile was previously reported for antimicrobial activity [61]. Derivatives of 1,2,4-Triazol [1,5-a] pyrimidine,5,7-dimethyl-2-phenyl and 4-Dichloromethyl-5 6-epoxy-2-methoxy-4-methyl-2-cyclohexenone were also reported for antifungal and phyto-pathogenic activity [62]. Among the other predicted compounds Vinylbital, Bruceantin, Digitoxigenin are already enlisted as important drugs.

One of the major criteria of the study was to isolate an actinobacteria having broad spectrum of antimicrobial property. In this study strain SMS_SU21 fulfilled this criterion most appropriately as it produced zone of inhibition >20 mm for more than half of the test organisms with a relatively low MIC value. GC-MS data and antioxidant property also supported that SMS_SU21 was the most active strain.

In the condition optimization study it was evident that cultivation in modified CSM media for eight days at 28 °C temperature with a shaking speed of 150 rpm is optimum for SMS_SU21 to produce the bioactive compounds.

## Conclusion

Nature being a sophisticated, versatile and energetic combinatorial chemist designs unique roadmap with infinite number of different and unpredictable ways, a series of exotic and effective structures, which have ever been made in laboratories. These actinobacteria from marine mangrove origin are priceless natural resources, having immense biosynthetic potential to be effective in biotechnological and industrial point of view. The secondary metabolites may be evolved in nature as some kind of response to the interplay of microbial genetics and natural product chemistry. Therefore isolation and purification of economically important secondary metabolites from actinomycetes of Sundarban mangrove and characterization of the bioactive compounds is a challenging solution for exploring antimicrobial and cytotoxic compounds from natural sources. Thus our study brings forward a good promise for future drug development and agricultural programs.

## Additional file

Additional file 1: Table S1. Different culture media with supplemented NaCl salt concentration (w/v) for isolation of actinobacteria. **Table S2.** Sporechain characteristic of nine actinomycetes as studied by scanning electron microscopy and results of the biochemical tests. **Table S3.** Colony characteristics of the isolates in different media.G- Growth, A-Aerial mycelium, R-Reverse colour, SP-Soluble pigment. **Table S4.** Biolog assay for nine selected strains. 71 carbon sources and 23 chemical utilization characteristics were examined in this study. Presence of a positive reaction was assigned the binary value 1, and negative reaction was assigned a binary value of 0. For each of the biochemical characteristics, at least one strain differed in any one characteristic. **Table S5.** Fatty acid methyl ester (FAME) analysis (%) for nine actinomycetes. **Table S6.** Results of the identity analysis of isolated actinobacteria strains based on 16S rRNA gene sequence performed on the EzTaxon server (last accessed on 05/06/2015). **Table S7.** Antimicrobial activity Comparison between SMS_SU21 crude extract and the HPLC purified fraction SMS_SU21-C. **Table S8.** Characteristics of the peaks obtained by GC-MS analysis. **Figure S1.** Colony morphology of the nine selected strains on seven different medium: Right-aerial mycelium, Left-substrate mycelium. **Figure S2.** a) HPLC chromatograph of SMS-SU21-crude extract.SMS_SU21C is the bioactive peak. b) TLC profile of the active crude (Viewed under UV 254 nm). (DOCX 6739 kb)
