# Supplementary material for: Antimicrobial activities of actinomycetes isolated from unexplored regions of Sundarbans mangrove ecosystem
Source: BMC Microbiol. 2015 Aug 21;15:170. doi: 10.1186/s12866-015-0495-4 (PMC4546244; doi:10.1186/s12866-015-0495-4)
Supplement: Additional file 1: Table S1. — Different culture media with supplemented NaCl salt concentration (w/v) for isolation of actinobacteria. Table S2. Sporechain characteristic of nine actinomycetes as studied by scanning electron microscopy and results of the biochemical tests. Table S3. Colony characteristics of the isolates in different media.G- Growth, A-Aerial mycelium, R-Reverse colour, SP-Soluble pigment. Table S4. Biolog assay for nine selected strains. 71 carbon sources and 23 chemical utilization characteristics were examined in this study. Presence of a positive reaction was assigned the binary value 1, and negative reaction was assigned a binary value of 0. For each of the biochemical characteristics, at least one strain differed in any one characteristic. Table S5. Fatty acid methyl ester (FAME) analysis (%) for nine actinomycetes. Table S6. Results of the identity analysis of isolated actinobacteria strains based on 16S rRNA gene sequence performed on the EzTaxon server (last accessed on 05/06/2015). Table S7. Antimicrobial activity Comparison between SMS_SU21 crude extract and the HPLC purified fraction SMS_SU21-C. Table S8. Characteristics of the peaks obtained by GC-MS analysis. Figure S1. Colony morphology of the nine selected strains on seven different medium: Right-aerial mycelium, Left-substrate mycelium. Figure S2. a) HPLC chromatograph of SMS-SU21-crude extract.SMS_SU21C is the bioactive peak. b) TLC profile of the active crude (Viewed under UV 254 nm). (DOCX 6739 kb) [file 12866_2015_495_MOESM1_ESM.docx]

**Additional file 1:**

**Table S1** Different culture media with supplemented NaCl salt concentration (w/v) for isolation of actinobacteria

|  | Number of isolate obtained in basal media supplemented with NaCl salt | | | | | | |
| --- | --- | --- | --- | --- | --- | --- | --- |
| Media used in this study | NaCl (0%) | NaCl (3%) | NaCl (6%) | NaCl (9%) | NaCl (12%) | NaCl (15%) | NaCl (18%) |
| M2 | 0 | 10  **(SMS_9)** | 2 | 0 | 1 | 0 | 0 |
| ISP4 | 0 | 7  **(SMS_5)** | 4  **(SMS_7)** | 0 | 0 | 0 | 0 |
| ISP2 | 0 | 6  **(SMS_13)** | 0 | 0 | 0 | 0 | 0 |
| GYM | 1 | 0 | 1 | 0 | 0 | 0 | 0 |
| Starch-Casein media | 0 | 4  **(SMS_10)** | 0 | 0 | 0 | 0 | 0 |
| IM8 | 0 | 2 | 0 | 0 | 0 | 0 | 0 |
| Cross streak media | 0 | 11  **(SMS_B,SMS_SU21,SMS_SU13)** | 3  **(SMS_SU23**) | 2 | 0 | 0 | 0 |

N.B. Most promising active strains are mentioned in boldface

**Table S2** Sporechain characteristic of nine actinomycetes as studied by scanning electron microscopy and results of the biochemical tests.

| **Parameters** | **SMS_B** | **SMS_5** | **SMS_7** | **SMS_9** | **SMS_10** | **SMS_13** | **SMS_SU13** | **SMS_SU21** | **SMS_SU23** |
| --- | --- | --- | --- | --- | --- | --- | --- | --- | --- |
| Spore Chain Ornamentation | Spiral | Reticulum–apertum | Straight | Spiral | Fascicled | Branched Reticulum–apertum | Straight | Reticulum–apertum with open loops | Reticulum–apertum |
| Spore Sizes (LXW) μm | 1.41X0.63 | 1.14X0.71 | 1.66X0.65 | 1.22X0.69 | 1.64X2.24 | 0.84X0.61 | 1.23X0.71 | 1.05X0.62 | 1.33X0.8 |
| Spore Surface | spiny | spiny | smooth | hairy | Warty | Smooth | spiny | Smooth | Smooth |
| Physiological Characterization Gram Reaction | + | + | + | + | + | + | + | + | + |
| Starch Hydrolysis | + | + | + | + | + | + | + | + | + |
| Casein Hydrolysis | + | + | + | + | + | + | + | + | + |
| Catalase Test | + | + | + | + | + | + | + | + | + |
| Nitrate Reduction | + | + | + | _ | + | + | + | + | + |
| Oxidase Reduction | _ | _ | _ | _ | _ | _ | _ | _ | _ |
| Hydrogen Sulphite Production | _ | _ | + | _ | _ | _ | _ | + | _ |
| Citrate Test | + | - | - | - | - | - | - | + | - |
| Urease Test | + | _ | _ | + | _ | + | + | + | _ |

**Table S3. Colony characteristics of the isolates in different media.G- Growth, A-Aerial mycelium, R-Reverse colour, SP-Soluble pigment.**

| **Media** | **SMS-B** | **SMS_5** | **SMS_7** | **SMS_9** | **SMS_10** | **SMS_13** | **SMS_SU13** | **SMS_SU21** | **SMS_SU23** |
| --- | --- | --- | --- | --- | --- | --- | --- | --- | --- |
| M2 | \| G  Good \| R  Tarpaulin  Grey \| \| --- \| --- \| \| A Green Beige \| SP  None \| | \| G  Good \| R  Oyster White \| \| --- \| --- \| \| A Oyster White \| SP  None \| | \| G  Good \| R  Clay Brown \| \| --- \| --- \| \| A Traffic Orange \| SP  None \| | \| G  Good \| R  Concrete Grey \| \| --- \| --- \| \| A Telegrey 4 \| SP  None \| | \| G  Good \| R  Signal Orange \| \| --- \| --- \| \| A Light Grey \| SP  None \| | \| G  Good \| R  Grey Beige \| \| --- \| --- \| \| A Green Beige \| SP  None \| | \| G  Good \| R  Grey Beige \| \| --- \| --- \| \| A Light Grey \| SP  None \| | \| G  Good \| R  Green Beige \| \| --- \| --- \| \| A Green Beige \| SP  None \| | \| G  Good \| R  Oyster White \| \| --- \| --- \| \| A Yellow Orange \| SP  None \| |
| ISP4 | \| G  Good \| R  Concrete  Grey \| \| --- \| --- \| \| A Green Beige \| SP  None \| | \| G  Good \| R  Light Pink \| \| --- \| --- \| \| A Oyster White \| SP  None \| | \| G  Good \| R  Signal Violet \| \| --- \| --- \| \| A Black Red \| SP  None \| | \| G  Good \| R  Grey  Aluminum \| \| --- \| --- \| \| A Anthracite  Grey \| SP  None \| | \| G  Good \| R  Green Beige \| \| --- \| --- \| \| A Squirrel Grey \| SP  None \| | \| G  Good \| R  Signal Grey \| \| --- \| --- \| \| A Umbra Grey \| SP  None \| | \| G  Good \| R  Mouse Grey \| \| --- \| --- \| \| A Grey White \| SP  None \| | \| G  Good \| R  Beige Grey \| \| --- \| --- \| \| A Grey White \| SP  None \| | \| G  Good \| R  Ruby Red \| \| --- \| --- \| \| A Orient Red \| SP  None \| |
| ISP2 | \| G  Good \| R  Signal  Yellow \| \| --- \| --- \| \| A Olive Grey \| SP  None \| | \| G  Good \| R  Melon Yellow \| \| --- \| --- \| \| A Ivory \| SP  None \| | \| G  Good \| R  Signal Yellow \| \| --- \| --- \| \| A Saffron Yellow \| SP  None \| | \| G  Good \| R  Chrome Yellow \| \| --- \| --- \| \| A Light Grey \| SP  None \| | \| G  Good \| R  Yellow Grey \| \| --- \| --- \| \| A Ochre  Brown \| SP  red \| | \| G  Good \| R  Honey Yellow \| \| --- \| --- \| \| A Ochre  Brown \| SP  None \| | \| G  Good \| R  Traffic Orange \| \| --- \| --- \| \| A Green Beige \| SP  None \| | \| G  Good \| R  Traffic Yellow \| \| --- \| --- \| \| A Telegrey 4 \| SP  None \| | \| G  Good \| R  Deep Orange \| \| --- \| --- \| \| Beige \| SP  None \| |
| GYM | \| G  Good \| R  Silk Grey \| \| --- \| --- \| \| A Window  Grey \| SP  None \| | \| G  Good \| R  Oyster White \| \| --- \| --- \| \| A Silk Grey \| SP  None \| | \| G  Good \| R  Light Ivory \| \| --- \| --- \| \| A Oyster White \| SP  None \| | \| G  Good \| R  Ivory \| \| --- \| --- \| \| A Light Ivory \| SP  None \| | \| G  Good \| R  Strawberry Red \| \| --- \| --- \| \| A Rose \| SP  None \| | \| G  Good \| R  Light Ivory \| \| --- \| --- \| \| A Olive Grey \| SP  None \| | \| G  Good \| R  Pebble Grey \| \| --- \| --- \| \| A Ivory \| SP  None \| | \| G  Good \| R  Oyster White \| \| --- \| --- \| \| A Ivory \| SP  None \| | \| G  Good \| R Oyster White \| \| --- \| --- \| \| A Oyster White \| SP  None \| |
| Starch-Casein | \| G  Good \| R  Signal Orange \| \| --- \| --- \| \| A Grey Beige \| SP  None \| | \| G  Good \| R  Deep Orange \| \| --- \| --- \| \| A Signal  White \| SP  None \| | \| G  Good \| R  Golden Yellow \| \| --- \| --- \| \| A Ivory \| SP  None \| | \| G  Good \| R  Traffic Yellow \| \| --- \| --- \| \| A Orange  Brown \| SP  None \| | \| G  Good \| R  Salmon Orange \| \| --- \| --- \| \| A Green Beige \| SP  None \| | \| G  Good \| R  Yellow Orange \| \| --- \| --- \| \| A Window  Grey \| SP  None \| | \| G  Good \| R  Golden Yellow \| \| --- \| --- \| \| A Light Grey \| SP  None \| | \| G  Good \| R  Pastel Orange \| \| --- \| --- \| \| A Green Beige \| SP  None \| | \| G  Good \| R  Green Beige \| \| --- \| --- \| \| A Green Beige \| SP  None \| |
| IM8 | \| G  Good \| R  Telegrey 1 \| \| --- \| --- \| \| A Telegrey4 \| SP  None \| | \| G  Good \| R  Sand Yellow \| \| --- \| --- \| \| A Traffic Yellow \| SP  None \| | \| G  Good \| R  Chrome Yellow \| \| --- \| --- \| \| A Beige \| SP  None \| | \| G  Good \| R  Saffron Yellow \| \| --- \| --- \| \| A Traffic Yellow \| SP  None \| | \| G  Good \| R  Traffic Yellow \| \| --- \| --- \| \| A Pastel Yellow \| SP  None \| | \| G  Good \| R  Telegrey 1 \| \| --- \| --- \| \| A White  Aluminum \| SP  None \| | \| G  Good \| R  Grey White \| \| --- \| --- \| \| A Grey White \| SP  None \| | \| G  Good \| R  Grey White \| \| --- \| --- \| \| A Telegrey 4 \| SP  None \| | \| G  Good \| R  Telegrey 2 \| \| --- \| --- \| \| A Grey White \| SP  None \| |
| CSM | \| G  Good \| R  Saffron Yellow \| \| --- \| --- \| \| A Light Grey \| SP  None \| | \| G  Good \| R  Oyster White \| \| --- \| --- \| \| A Pebble Grey \| SP  None \| | \| G  Good \| R  Oyster White \| \| --- \| --- \| \| A Yellow Grey \| SP  None \| | \| G  Good \| R  Ivory \| \| --- \| --- \| \| A Green  Brown \| SP  None \| | \| G  Good \| R  Ivory \| \| --- \| --- \| \| A Ochre  Brown \| SP  None \| | \| G  Good \| R  Cream \| \| --- \| --- \| \| A Ochre  Brown \| SP  None \| | \| G  Good \| R  Saffron Yellow \| \| --- \| --- \| \| A Light Grey \| SP  None \| | \| G  Good \| R  Yellow Orange \| \| --- \| --- \| \| A Telegrey 1 \| SP  None \| | \| G  Good \| R  Oyster White \| \| --- \| --- \| \| A Maize Yellow \| SP  None \| |

**Table S4. Biolog assay for nine selected strains. 71 carbon sources and 23 chemical utilization characteristics were examined in this study. Presence of a positive reaction was assigned the binary value 1, and negative reaction was assigned a binary value of 0. For each of the biochemical characteristics, at least one strain differed in any one characteristic.**

| **Properties** | **SMS_B** | **SMS_5** | **SMS_7** | **SMS_9** | **SMS_10** | **SMS_13** | **SMS_SU13** | **SMS_SU21** | **SMS_SU23** |
| --- | --- | --- | --- | --- | --- | --- | --- | --- | --- |
| Dextrin | 1 | 0 | 1 | 1 | 0 | 1 | 1 | 1 | 1 |
| D-Maltose | 1 | 1 | 0 | 0 | 1 | 1 | 1 | 1 | 1 |
| D-Trehalose | 1 | 0 | 0 | 1 | 0 | 1 | 1 | 0 | 1 |
| D-Cellobiose | 1 | 1 | 0 | 0 | 1 | 1 | 1 | 0 | 1 |
| Gentiobiose | 1 | 1 | 0 | 0 | 0 | 1 | 0 | 0 | 1 |
| Sucrose | 1 | 1 | 0 | 0 | 1 | 1 | 0 | 0 | 1 |
| D-Turanose | 1 | 1 | 0 | 0 | 1 | 1 | 0 | 0 | 1 |
| Stachyose | 0 | 0 | 0 | 0 | 1 | 1 | 0 | 0 | 1 |
| pH 6 | 1 | 1 | 1 | 1 | 1 | 1 | 1 | 1 | 1 |
| pH 5 | 1 | 1 | 1 | 1 | 1 | 1 | 1 | 1 | 1 |
| D-Raffinose | 0 | 0 | 0 | 0 | 1 | 0 | 0 | 0 | 1 |
| α-D-Lactose | 1 | 1 | 1 | 0 | 1 | 1 | 0 | 0 | 1 |
| D-Melibiose | 1 | 1 | 0 | 1 | 1 | 1 | 1 | 1 | 1 |
| β-Methyl-Dglucoside | 1 | 0 | 0 | 1 | 1 | 1 | 1 | 1 | 1 |
| D-Salicin | 1 | 0 | 0 | 1 | 1 | 1 | 1 | 1 | 1 |
| N-Acetyl-DGlucosamine | 1 | 1 | 0 | 0 | 1 | 1 | 1 | 1 | 1 |
| N-Acetyl-β-DMannosamine | 1 | 0 | 0 | 1 | 0 | 1 | 1 | 0 | 0 |
| N-Acetyl-DGalactosamine | 1 | 0 | 0 | 0 | 1 | 1 | 0 | 0 | 0 |
| N-Acetyl NeuraminicAcid | 0 | 1 | 0 | 1 | 1 | 1 | 0 | 0 | 1 |
| 1% NaCl | 1 | 1 | 1 | 1 | 1 | 1 | 1 | 1 | 1 |
| 4% NaCl | 1 | 1 | 1 | 1 | 1 | 0 | 1 | 1 | 1 |
| 8% NaCl | 1 | 1 | 1 | 1 | 1 | 0 | 1 | 1 | 1 |
| α-D-Glucose | 1 | 1 | 1 | 1 | 0 | 1 | 1 | 1 | 1 |
| D-Mannose | 1 | 1 | 1 | 0 | 0 | 0 | 0 | 0 | 1 |
| D-Fructose | 1 | 1 | 0 | 1 | 1 | 1 | 0 | 0 | 1 |
| D-Galactose | 1 | 1 | 0 | 0 | 0 | 1 | 0 | 0 | 1 |
| 3-Methyl Glucose | 0 | 0 | 0 | 0 | 0 | 0 | 0 | 0 | 0 |
| D-Fucose | 1 | 0 | 1 | 0 | 1 | 1 | 1 | 0 | 0 |
| L-Fucose | 1 | 1 | 0 | 0 | 1 | 1 | 0 | 0 | 0 |
| L-Rhamnose | 1 | 1 | 0 | 0 | 1 | 1 | 0 | 0 | 1 |
| Inosine | 1 | 1 | 1 | 1 | 1 | 1 | 1 | 1 | 1 |
| 1% Sodium Lactate | 1 | 1 | 1 | 1 | 1 | 1 | 1 | 1 | 1 |
| Fusidic Acid | 0 | 1 | 0 | 0 | 0 | 0 | 0 | 0 | 0 |
| D-Serine | 1 | 1 | 1 | 1 | 1 | 1 | 1 | 1 | 1 |
| D-Sorbitol | 0 | 0 | 1 | 0 | 1 | 0 | 0 | 0 | 0 |
| D-Mannitol | 1 | 1 | 0 | 0 | 1 | 1 | 0 | 0 | 1 |
| D-Arabitol | 1 | 1 | 0 | 1 | 1 | 1 | 0 | 0 | 1 |
| myo-Inositol | 1 | 1 | 0 | 0 | 0 | 1 | 0 | 0 | 1 |
| Glycerol | 1 | 1 | 0 | 0 | 1 | 1 | 1 | 1 | 1 |
| D-Glucose-6-PO4 | 1 | 1 | 1 | 1 | 1 | 1 | 1 | 0 | 1 |
| D-Fructose-6-PO4 | 1 | 1 | 0 | 1 | 1 | 1 | 0 | 1 | 0 |
| D-Aspartic Acid | 1 | 1 | 0 | 0 | 1 | 1 | 0 | 0 | 1 |
| D-Serine | 1 | 1 | 0 | 0 | 0 | 1 | 0 | 0 | 0 |
| Troleandomycin | 0 | 1 | 0 | 0 | 0 | 0 | 0 | 0 | 0 |
| Rifamycin SV | 1 | 1 | 1 | 1 | 1 | 0 | 0 | 1 | 1 |
| Minocycline | 0 | 1 | 1 | 0 | 0 | 0 | 0 | 0 | 0 |
| Gelatin | 1 | 1 | 1 | 1 | 1 | 1 | 1 | 1 | 1 |
| Glycyl-L-Proline | 1 | 1 | 1 | 1 | 0 | 1 | 1 | 1 | 1 |
| L-Alanine | 1 | 1 | 0 | 1 | 0 | 1 | 1 | 1 | 1 |
| L-Arginine | 1 | 1 | 0 | 1 | 1 | 1 | 1 | 1 | 1 |
| L-Aspartic Acid | 1 | 1 | 0 | 1 | 1 | 1 | 1 | 1 | 1 |
| L-Glutamic Acid | 1 | 1 | 0 | 1 | 1 | 1 | 1 | 1 | 1 |
| L-Histidine | 1 | 1 | 0 | 1 | 1 | 1 | 1 | 1 | 1 |
| L-Pyroglutamic Acid | 1 | 1 | 0 | 0 | 1 | 1 | 1 | 1 | 1 |
| L-Serine | 1 | 1 | 0 | 1 | 1 | 1 | 1 | 1 | 1 |
| Lincomycin | 0 | 1 | 0 | 0 | 0 | 0 | 1 | 0 | 1 |
| Guanidine HCl | 1 | 0 | 1 | 1 | 1 | 1 | 1 | 1 | 1 |
| Niaproof 4 | 0 | 1 | 0 | 0 | 1 | 0 | 0 | 0 | 1 |
| Pectin | 1 | 0 | 1 | 0 | 0 | 1 | 1 | 1 | 0 |
| D-Galacturoni Acid | 1 | 0 | 1 | 0 | 1 | 1 | 0 | 0 | 0 |
| L-Galactonic Acid Lactone | 0 | 0 | 0 | 0 | 1 | 0 | 0 | 0 | 0 |
| D-Gluconic Acid | 1 | 1 | 0 | 1 | 1 | 1 | 1 | 1 | 1 |
| D-Glucuronic Acid | 1 | 1 | 0 | 1 | 1 | 1 | 1 | 1 | 1 |
| Glucuronamide | 1 | 1 | 1 | 1 | 1 | 1 | 1 | 1 | 0 |
| Mucic Acid | 1 | 0 | 0 | 0 | 1 | 1 | 0 | 0 | 0 |
| Quinic Acid | 1 | 1 | 1 | 0 | 1 | 1 | 1 | 1 | 1 |
| D-Saccharic Acid | 1 | 0 | 0 | 1 | 1 | 1 | 0 | 1 | 0 |
| Vancomycin | 0 | 1 | 0 | 0 | 0 | 0 | 1 | 1 | 0 |
| Tetrazolium Violet | 0 | 1 | 1 | 0 | 0 | 0 | 0 | 0 | 0 |
| Tetrazolium Blue | 0 | 1 | 0 | 0 | 0 | 0 | 0 | 0 | 1 |
| p-Hydroxy-Phenylacetic Acid | 0 | 0 | 0 | 0 | 0 | 0 | 0 | 0 | 0 |
| Methyl Pyruvate | 1 | 0 | 1 | 0 | 1 | 1 | 1 | 1 | 1 |
| D-Lactic Acid Methyl Ester | 1 | 0 | 0 | 1 | 1 | 1 | 0 | 0 | 1 |
| L-Lactic Acid | 1 | 1 | 0 | 1 | 1 | 1 | 0 | 0 | 1 |
| Citric Acid | 1 | 0 | 0 | 1 | 1 | 1 | 1 | 1 | 1 |
| α-Keto-Glutaric Acid | 1 | 0 | 0 | 1 | 1 | 1 | 0 | 1 | 0 |
| D-Malic Acid | 1 | 1 | 0 | 0 | 1 | 1 | 1 | 1 | 0 |
| L-Malic Acid | 1 | 1 | 0 | 1 | 1 | 1 | 1 | 1 | 1 |
| Bromo-Succinic Acid | 0 | 1 | 0 | 1 | 1 | 0 | 1 | 1 | 0 |
| Nalidixic Acid | 1 | 1 | 1 | 1 | 1 | 1 | 1 | 1 | 1 |
| Lithium Chloride | 1 | 1 | 1 | 1 | 1 | 0 | 1 | 1 | 1 |
| Potassium Tellurite | 1 | 1 | 1 | 1 | 1 | 1 | 1 | 1 | 0 |
| Tween 40 | 0 | 0 | 1 | 0 | 0 | 0 | 1 | 1 | 1 |
| γ-Amino-Butryric Acid | 1 | 1 | 1 | 1 | 1 | 0 | 1 | 1 | 1 |
| α-Hydroxy- Butyric Acid | 1 | 1 | 0 | 1 | 1 | 0 | 1 | 1 | 0 |
| β-Hydroxy-D,Lbutyric Acid | 1 | 1 | 0 | 1 | 0 | 1 | 1 | 0 | 0 |
| α-Keto-Butyric Acid | 1 | 1 | 0 | 1 | 0 | 1 | 1 | 1 | 0 |
| Acetoacetic Acid | 1 | 1 | 1 | 1 | 1 | 1 | 1 | 1 | 0 |
| Propionic Acid | 1 | 1 | 1 | 1 | 1 | 1 | 1 | 1 | 0 |
| Acetic Acid | 1 | 1 | 1 | 0 | 1 | 1 | 1 | 1 | 0 |
| Formic Acid | 1 | 1 | 0 | 1 | 1 | 1 | 0 | 0 | 0 |
| Aztreonam | 1 | 1 | 1 | 1 | 1 | 0 | 1 | 1 | 0 |
| Sodium Butyrate | 1 | 1 | 1 | 1 | 1 | 1 | 1 | 1 | 0 |
| Sodium Bromate | 1 | 1 | 1 | 1 | 1 | 0 | 1 | 0 | 0 |

**Table S5 Fatty acid methyl ester (FAME) analysis (%) for nine actinomycetes.**

| **Fatty acid** | **SMS_SU13** | **SMS_SU21** | **SMS_5** | **SMS_7** | **SMS_9** | **SMS_10** | **SMS_13** | **SMS_23** | **SMS_B** |
| --- | --- | --- | --- | --- | --- | --- | --- | --- | --- |
| 11:0 ISO | NA | NA | 0.07 | NA | NA | NA | 0.02 | NA | NA |
| 11:0 ANTEISO | NA | NA | 0.11 | 0.15 | NA | NA | 0.04 | NA | NA |
| 11:0 | NA | NA | NA | NA | NA | NA | 0.02 | NA | NA |
| 12:0 ISO | 0.12 | 0.08 | 0.16 | 0.15 | 0.13 | 0.19 | 0.25 | 0.07 | 0.05 |
| 12:0 | 0.16 | 0.10 | 0.20 | 0.21 | 0.26 | 0.14 | 0.12 | 0.20 | 0.16 |
| 11:0 ISO 3OH | 0.90 | 0.05 | 0.26 | 0.24 | 0.72 | 0.41 | 0.10 | 0.06 | 0.27 |
| 13:0 ISO | 0.30 | 0.27 | 0.46 | 0.40 | 0.22 | 0.35 | 0.22 | 0.30 | 0.28 |
| 13:0 ANTEISO | 0.15 | 0.19 | 0.62 | 0.53 | 0.34 | 0.44 | 0.34 | 0.28 | 0.15 |
| 13:1 AT 12-13 | NA | NA | NA | NA | NA | NA | 0.04 | NA | NA |
| 13:0 | NA | 0.08 | 0.14 | 0.14 | NA | 0.06 | 0.13 | 0.12 | 0.09 |
| 14:1 ISO E | NA | NA | NA | NA | NA | NA | 0.07 | NA | NA |
| 14:0 ISO | **3.72** | **3.66** | **4.21** | **3.83** | **3.75** | **5.64** | **7.40** | 6.10 | 3.63 |
| 14:0 | 0.27 | 0.35 | 0.80 | 0.94 | 0.29 | 0.30 | 1.26 | 1.06 | 0.25 |
| SUM IN FEATURE 1 | 0.12 | 0.06 | 0.06 | 0.05 | 0.13 | 0.12 | 0.05 | 0.09 | 0.11 |
| SUM IN FEATURE 2 | NA | NA | NA | NA | NA | NA | 0.07 | NA | NA |
| 15:0 ISO | **14.83** | **13.68** | **15.64** | **14.63** | **13.18** | **15.25** | **7.64** | **9.12** | **13.95** |
| 15:0 ANTEISO | **16.98** | **19.19** | **22.41** | **22.25** | **23.58** | **20.60** | **14.18** | **22.48** | **16.28** |
| 15:1 B | 0.20 | 0.24 | 0.12 | 0.09 | 0.25 | 0.17 | 0.74 | NA | 0.14 |
| 15:0 | 2.12 | 2.86 | 2.60 | 2.63 | 2.31 | 2.18 | **4.03** | 2.36 | 2.28 |
| 14:0 ISO 3OH | NA | NA | NA | NA | NA | NA | 0.04 | NA | NA |
| 16:1 ISO H | **1.76** | 1.97 | 0.93 | 1.03 | 1.38 | 1.92 | 1.48 | 0.74 | 1.65 |
| 16:0 ISO | **19.82** | **25.65** | **17.46** | **16.45** | **21.13** | **23.01** | **33.13** | **20.46** | **22.18** |
| 16:1 CIS 9 | **2.30** | 2.64 | 1.72 | 1.56 | 1.14 | 1.41 | **5.71** | 2.36 | 2.12 |
| 16:1 C | **NA** | NA | NA | NA | NA | NA | **0.05** | NA | NA |
| 16:0 | **5.16** | **5.28** | **10.88** | 11.79 | **6.19** | **5.67** | **8.56** | **15.76** | **5.31** |
| 15:0 20H | **NA** | **NA** | **NA** | NA | **NA** | **NA** | 0.05 | NA | NA |
| 16:0 ISO 10METHYL | **NA** | NA | **NA** | O.11 | NA | NA | NA | NA | NA |
| 16:0 9? METHYL | **5.16** | 4.47 | 2.38 | 2.44 | 2.27 | 2.68 | 0.91 | 1.05 | 5.31 |
| 17:1 ANTEISO C | **1.77** | 2.11 | 1.14 | 1.44 | 1.36 | 1.40 | 0.76 | 1.36 | 1.86 |
| 17:0 ISO | **9.30** | **5.58** | **5.58** | **5.44** | **5.99** | **7.00** | **2.19** | **3.28** | **9.00** |
| 17:0 ANTEISO | **10.12** | **9.71** | **8.37** | **9.09** | **11.04** | **8.68** | **6.25** | **9.28** | **10.42** |
| 17:1 CIS 9 | 0.99 | 0.76 | 0.42 | 0.40 | 0.27 | 0.44 | 0.68 | 0.38 | 1.16 |
| 17:0 CYCLO | 0.49 | 0.34 | 0.84 | 1.19 | 0.13 | 0.49 | 0.80 | 0.78 | 0.43 |
| 17:0 | **1.48** | 1.03 | 1.14 | 1.15 | 0.79 | 0.82 | 1.06 | 1.37 | 1.48 |
| 16:0 ISO 30H | **NA** | NA | NA | NA | NA | NA | 0.35 | NA | NA |
| 17:0 10METHYL | 0.13 | 0.14 | NA | NA | NA | NA | 0.05 | NA | 0.12 |
| 16:0 3OH | NA | NA | NA | NA | NA | NA | 0.23 | NA | NA |
| Unknown 17.595 sm | NA | 0.06 | 0.29 | 0.45 | NA | NA | 0.11 | NA | 0.18 |
| 18:1 ISO H | 0.38 | NA | NA | NA | NA | NA | NA | NA | NA |
| 18:0 ISO | 0.47 | 0.11 | 0.12 | 0.13 | 0.21 | NA | 0.26 | 0.16 | 0.21 |
| 18:1 CIS 9 | NA | 0.07 | 0.11 | 0.14 | 0.50 | NA | 0.09 | NA | 0.13 |
| 17:0 ISO 2OH | 0.11 | 0.05 | 0.16 | 0.20 | NA | NA | 0.09 | NA | 0.14 |
| 17:0 2OH | NA | NA | NA | NA | NA | NA | 0.10 | NA | NA |
| 18:0 | 0.28 | 0.10 | 0.2 | 0.42 | 1.26 | 0.36 | 0.19 | 0.54 | 0.29 |
| 19:1 TRANS 7 | 0.40 | NA | NA | 0.22 | 0.39 | 0.27 | NA | 0.12 | 0.22 |
| 19:0 ANTEISO | NA | 0.12 | NA | NA | 0.33 | NA | NA | NA | 0.16 |
| 19.0 | NA | NA | NA | NA | NA | NA | NA | NA | NA |
| 19.0 ISO | NA | NA | NA | NA | NA | NA | 0.04 | NA | NA |
| SUMMED FEATURE 1 | 0.12 | 0.06 | 0.06 | 0.05 | 0.13 | 0.12 | 0.05 | 0.09 | 0.11 |
| SUMMED FEATURE 7 | NA | 0.03 | 0.07 | 0.10 | 0.21 | NA | 0.03 | 0.11 | NA |
| SUMMED FEATURE 6 | NA | NA | NA | NA | 0.24 | NA | NA | NA | NA |
|  |  |  |  |  |  |  |  |  |  |

**Table S6 Results of the identity analysis of isolated actinobacteria strains based on 16S rRNA gene sequence performed**

**on the EzTaxon server (last accessed on 05/06/2015).**

| Actinobacterial strains | NCBI  Accession No. | Length (bp) | Closest species match | Similarity (%) |
| --- | --- | --- | --- | --- |
| SMS_B | KJ777668 | 1231 | *Streptomyces variabilis* NBRC 12825^T^ | 98.62 |
| SMS_5 | KJ777669 | 1235 | *Streptomyces tendae* ATCC 19812^T^ | 98.94 |
| SMS_7 | KJ777670 | 1233 | *Streptomyces tendae* ATCC 19812^T^ | 97.88 |
| SMS_9 | KJ777671 | 1230 | *Streptomyces atrovirens* NRRL B-16357^T^ | 98.21 |
| SMS_10 | KJ777672 | 1431 | *Streptomyces albogriseolus* NRRL B-1305^T^ | 93.57 |
| SMS_13 | KJ777673 | 1231 | *Streptomyces lusitanus* NBRC 13464^T^ | 98.77 |
| SMS_SU13 | KJ777674 | 1234 | *Streptomyces variabilis* NBRC 12825^T^ | 96.59 |
| SMS_SU21 | KJ777675 | 1223 | *Streptomyces griseorubens* NBRC 12780^T^ | 99.75 |
| SMS_SU23 | KJ777676 | 1231 | *Streptomyces coelicoflavus* NBRC 15399^T^ | 99.02 |

**Table S7.Antimicrobial activity Comparison between SMS_SU21 crude extract and the HPLC purified fraction SMS_SU21-C.**

| **Test organism** | **SMS_SU21-Crude extract**  **zone of inhibition in mm(20 μl/well)** | **Fraction SMS_SU21-Czone of inhibition in mm(20 μl/well)** |
| --- | --- | --- |
| *Vibrio cholera* (MTCC 3906) | 17±2 | 23±1 |
| *Staphylococcus aureus* (ATCC 25923) | 14.5±1 | 18±1.33 |

**Table S8** Characteristics of the peaks obtained by GC-MS analysis.

| **RT** | **Compound** | **activity** | **Theoretical Formula** | **MW** |
| --- | --- | --- | --- | --- |
| 9.89 | 3-Ethoxy-4-methoxyphenol | Antitumor activity against human HCT116 cells | C9H12O3 | 168 |
| 10.257 | trimethylboroxine | Neuropsychiatric drug | C_3_H_9_B_3_O_3_ | 126 |
| 10.547 | N-TFA-N-butyl-N-methylalanine ester | No activity | C_10_H_16_F_3_NO_4_ | 255 |
| 10.684 | Vinylbital | sedative hypnotic drug | C_11_H_16_N_2_O_3_ | 224 |
| 11.099 | 1-prolineN-allyloxycarbonyl-, octadecyl ester | No activity | C_27_H_49_NO_4_ | 451 |
| 11.222 | 5-propan-2-yl-5-prop-2-enyl-1,3-diazinane-2,4,6-trione(Aprobarbital) | sedative hypnotic drug | C_10_H_14_N_2_O_3_ | 210 |
| 11.271 | 2,6-dimethoxy phenol | Antioxidant | C_10_H_12_O_4_ | 196 |
| 11.295 | 2,2-propyl-N-ethylpiperidine | Antimicrobial activity | C_10_H_6_N_2_ | 154 |
| 11.395 | Bruceantin | Antitumor activity | C_28_H_38_0_11_ | 548 |
| 12.497 | 1-[(2-thienylcarbonyl)]-2,5-pyrrolidinedione | No activity | C_9_H_7_NO_4_S | 225 |
| 12.558 | 2-Chloro-N-isopropyl-N-phenylacetamide (Propachlor) | Herbicidal activity | C_11_H_14_ClNO | 211 |
| 12.970 | Thionine | No activity | C_12_H_10_N_3_S | 228 |
| 13.044 | Mefenamic acid | non-steroidal anti-inflammatory drug | C_15_H_15_NO_2_ | 241 |
| 13.736 | Lenacil | Pesticides | C_13_H_18_N_2_O_2_ | 234 |
| 13.884 | N-(7,7-dimethyl-2-oxobicyclo[2.2.1.]hept-1-yl)methanesulfonamide | No activity | C_10_H_17_NO_3_S | 231 |
| 14.084 | 4-Dichloromethyl-5 6-epoxy-2-methoxy-4-methyl-2-cyclohexenone | Antifungal activity | C_9_H_10_Cl_2_O_3_ | 236 |
| 14.523 | 1,2,4-Triazol[1,5-a]pyrimidine,5,7-dimethyl-2-phenyl | Phyto-phathogenic activity | C_13_H_12_N_4_ | 224 |
| 14.826 | 4-(2-Butylamino-1-hydroxyethyl)phenol (bamethan) | Vasodilator | C_12_H_19_NO_2_ | 209 |
| 15.7121 | Bicycle[4.1.0]hepta-1,3,5-triene,7,7-difluoro | No activity | C_7_H_4_F_2_ | 126 |
| 15.785 | 1,3-cyclopentanedione,2-isopentyl | Antifungal activity | C_10_H_16_O_2_ | 168 |
| 16.724 | 2,6-difluorobenzoic acid,4-nitrophenyl ester | No activity | C_13_H_6_BrF_3_O_2_ | 330 |
| 18.410 | Isoquinoline-1-carbonitrile | Antimicrobial and antifungal | C_10_H_6_N_2_ | 154 |
| 18.998 | Digitoxigenin | Cardenolidic activity | C_23_H_34_O_4_ | 374 |
| 19.416 | Cyclohexanone, 3-ethenyl-3-methyl-2-(1-methylethenyl)-6-(1-methylethylidene) | No activity | C_15_H_22_O | 218 |

**Fig. S1Colony morphology of the nine selected strains on seven different medium: Right-aerial mycelium, Left-substrate mycelium**

A-ISP_4,_ B-ISP_2,_ C-Starch Casein, D-CSM, E-IM_8,_ F-GYM, G-M_2_

1-SMS_B, 2-SMS_5, 3-SMS_9,4-SMS_10, 5-SMS_13, 6-SMS_SU13, 7-SMS_SU21, 8-SMS_SU21, SMS_SU7.


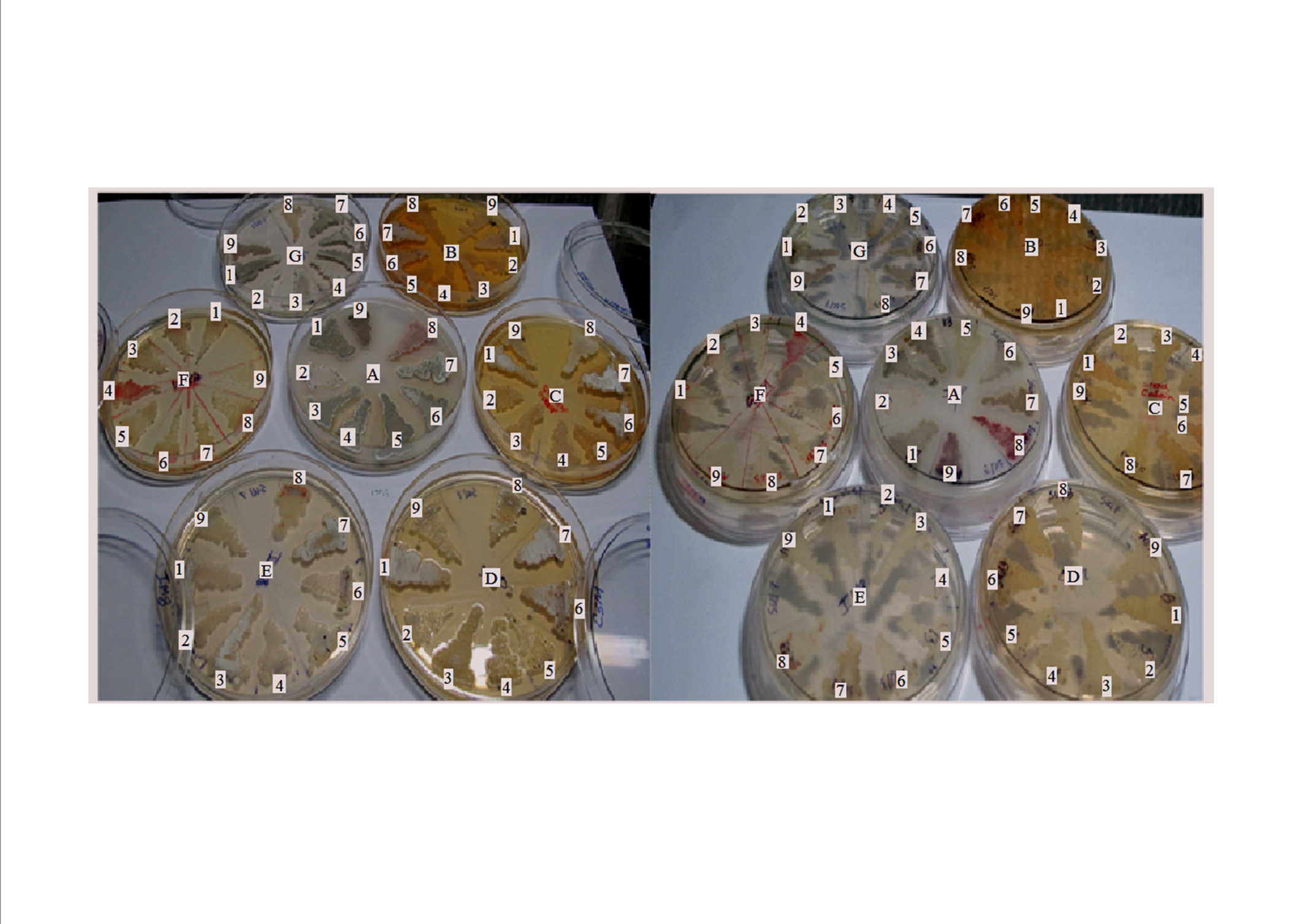


**Fig S2a)HPLC chromatograph of SMS-SU21-crude extract.SMS_SU21C is the bioactive peak.**b) **TLC profile of the active crude (Viewed under**

**UV 254 nm)**


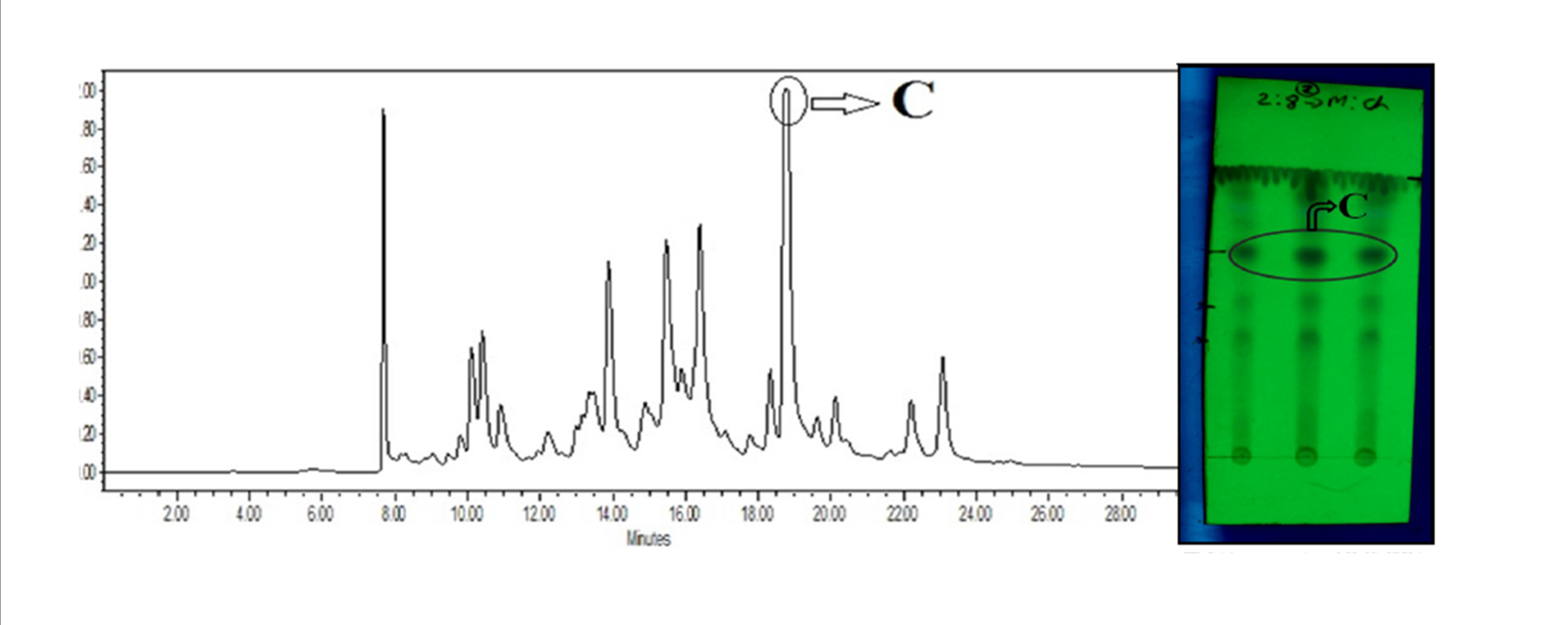


b

a
